# Pten-mediated Gsk3β modulates the naïve pluripotency maintenance in embryonic stem cells

**DOI:** 10.1038/s41419-020-2271-0

**Published:** 2020-02-07

**Authors:** Wuming Wang, Gang Lu, Xianwei Su, Chengcheng Tang, Hongjian Li, Zhiqiang Xiong, Chi-Kwan Leung, Man-Sze Wong, Hongbin Liu, Jin-Long Ma, Hoi-Hung Cheung, Hsiang-Fu Kung, Zi-Jiang Chen, Wai-Yee Chan

**Affiliations:** 1 CUHK-SDU Joint Laboratory on Reproductive Genetics, School of Biomedical Sciences, The Chinese University of Hong Kong, Hong Kong, China; 2 National Research Center for Assisted Reproductive Technology and Reproductive Genetics, Jinan, 250001 China; 3 SDIVF R&D Centre, Hong Kong Science Park, Shatin, Hong Kong, China; 4 Center for Reproductive Medicine, Renji Hospital, School of Medicine, Shanghai Jiao Tong University, Shanghai, 200135 China

**Keywords:** Cell signalling, Embryonic stem cells

## Abstract

Mouse embryonic stem cells (ESCs) are isolated from the inner cell mass of blastocysts, and they exist in different states of pluripotency—naïve and primed states. *Pten* is a well-known tumor suppressor. Here, we generated *Pten*^−/−^ mouse ESCs with the CRISPR-Cas9 system and verified that *Pten*^−/−^ ESCs maintained naïve pluripotency by blocking Gsk3β activity. Serum/LIF and 2i (MAPK and GSK3 inhibitors) conditions are commonly used for ESC maintenance. We show that the Pten-inhibitor SF1670 contributed to sustaining mouse ESCs and that Pten activation by the S380A, T382A, and T383A mutations (Pten-A3) suppressed the pluripotency of ESCs. The in vivo teratoma formation ability of SF1670-treated ESCs increased, while the Pten-A3 mutations suppressed teratoma formation. Furthermore, the embryoid bodies derived from *Pten*-deficient ESCs or SF1670-treated wild-type ESCs showed greater expression of ectoderm and pluripotency markers. These results suggest that Pten-mediated Gsk3β modulates the naïve pluripotency of ESCs and that *Pten* ablation regulates the lineage-specific differentiation.

## Introduction

Embryonic stem cells (ESCs) are pluripotent stem cells derived from the inner cell mass of the early mammalian embryo^1–3^. ESCs possess the ability to self-renew and to differentiate into cells representative of all three embryonic germ layers^4^. In both mice and humans, ESCs exist in different pluripotent states such as the naïve and primed states, and these two states of pluripotency are distinguished by unique molecular and cellular features^5^. Self-renewing, pluripotent, and lineage primed states have been defined by multiple methodologies, including the recently reported method of single-cell expression profiling^6^.

Small-molecule inhibitors have been used to maintain the pluripotency of mouse and primate ESCs^7,8^. Mouse ESCs are maintained in a ground state of pluripotency in the presence of MAP kinase/ERK kinase (MEK) and glycogen synthase kinase 3 (GSK3) inhibitors (2i, PD0325901 and CHIR99021) that can increase the expression of pluripotency factors in ESCs^9^, and the cytokine leukemia inhibitory factor (LIF) drives self-renewal of mouse ESCs by activating the transcription factor STAT3^10,11^. Wnt/β-catenin signaling is important in maintaining the pluripotency of mouse ESCs by inhibiting GSK3 with CHIR99021^7,9^. BIO (6-bromoindirubin-3′-oxime), a GSK3-specific pharmacological inhibitor, inhibits the differentiation of ESCs and activates the Wnt pathway to maintain the expression of the pluripotency sustaining factors *Rex1*, *Oct4*, and *Nanog*^7^.

The *Pten* gene is a well-known tumor suppressor and is essential for embryonic development. *Pten*^*−/−*^ homozygous embryos exhibit lethality with defective placentation and death by embryonic day (E) 7.5^12^. *Pten* controls the renewal and differentiation of neural and glioma stem/progenitor cells^13,14^, and *Pten*^*−/−*^ ESCs form aberrant embryoid bodies (EBs) and show an altered differentiation potential^15^. PTEN is a phosphatase that dephosphorylates phosphatidylinositol-3, 4, 5-trisphosphate (PIP_3_), and it negatively regulates the phosphoinositide-3 kinase (PI3K) signaling pathway to inhibit AKT activity^16^. PI3K signaling has recently been shown to manipulate mammalian preimplantation embryogenesis^17^, and inhibition or disruption of the PI3K/AKT pathway results in loss of pluripotency and viability of ESCs and promotes the differentiation of ESCs^18,19^.

In this study, we established the *Pten*^*−/−*^ and Pten-A3 mutant (S380A, T382A, and T383A) ESC lines by using the CRISPR-Cas9 system and aimed to determine the roles of *Pten* in modulating the naïve pluripotency maintenance of ESCs and to define the underlying molecular mechanism.

## Results

### Loss of tumor suppressor *Pten* maintains ESC pluripotency and modulates ESC differentiation

**Fig. 1:**
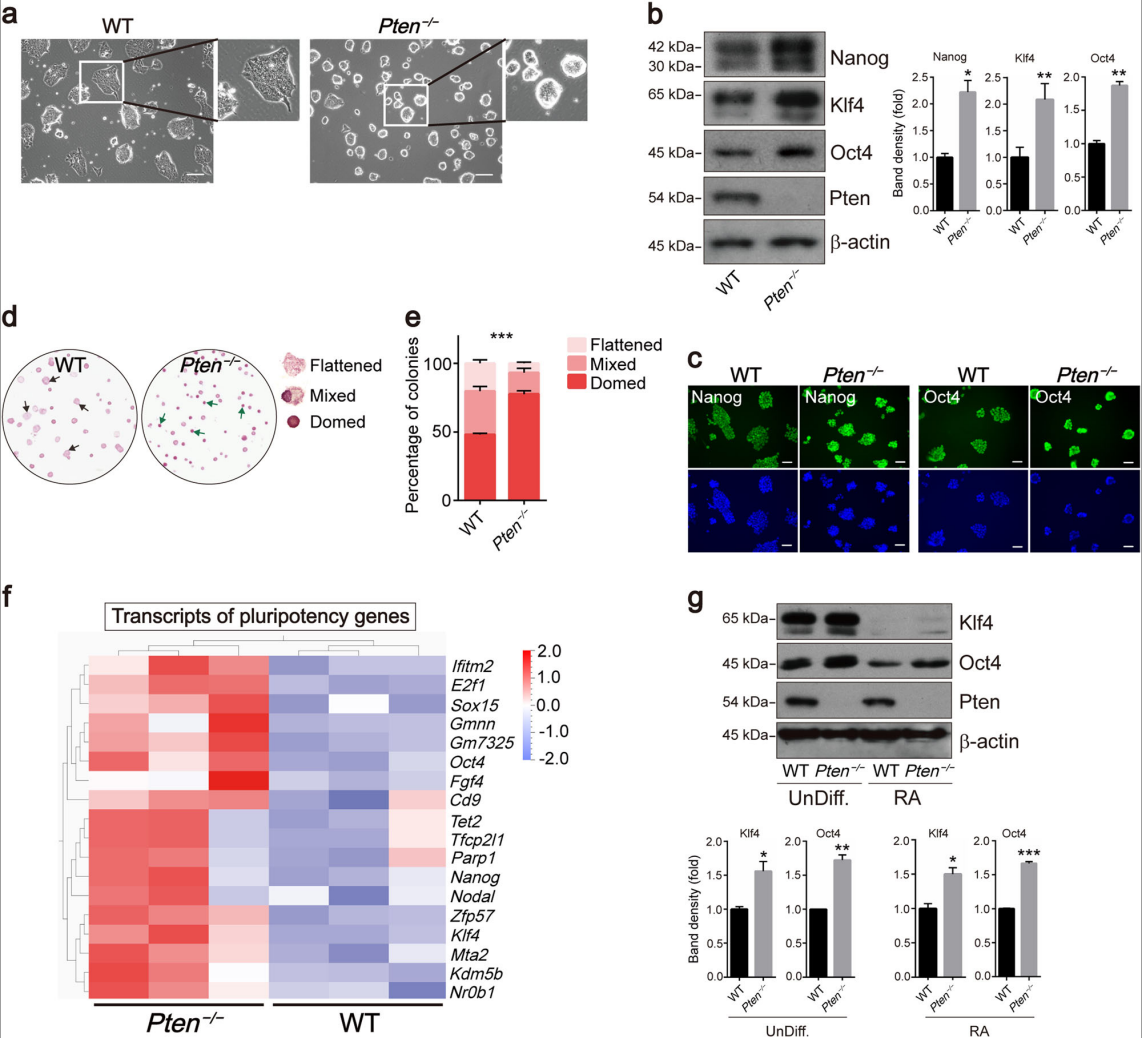
*Pten* deletion promotes the pluripotency of ESCs and suppresses early differentiation. **a** Phase-contrast images of wild-type (WT) and *Pten*^*−/−*^ ESCs. *Pten*^*−/−*^ ESCs had a greater proportion in the ground state. Scale bars, 100 µm. **b** Western blot analysis showed that the expression of pluripotency markers (*Nanog*, *Oct4*, and *Klf4*) was increased in *Pten*^*−/−*^ ESCs. **c** Immunofluorescence staining for Nanog and Oct4 in WT and *Pten*^*−/−*^ ESCs. DNA was stained with DAPI to indicate nuclei. Scale bars, 50 µm. **d** AP staining of WT and *Pten*^*−/−*^ ESC colonies cultured for 4 days. There are more flattened colonies (black arrows) in WT ESCs, and more domed colonies (green arrows) in *Pten*^*−/−*^ ESCs. Scale bars, 100 µm. **e** Analysis of colony morphology of WT and *Pten*^*−/−*^ ESCs. Error bars indicate mean ± SEM (*n* = 3), and 80 colonies were scored in each replicate. **f** Heat map of FPKM values of pluripotency genes in WT and *Pten*^*−/−*^ ESCs. The heat map was normalized with sigma-normalization per row. See also Table S2. **g** Western blot analysis of WT and *Pten*^*−/−*^ undifferentiated (UnDiff.) ESCs or treated with 200 nM RA for 48 h showing the expression of Klf4 and Oct4. β-actin was used as the loading control.

To explore the function of *Pten* in mouse ESCs, we generated *Pten*^*−/−*^ ESCs using the CRISPR-Cas9 system (Fig. S1a). These cell lines contained mutant alleles with small insertions or deletions (indels) at the target sites. We utilized clone G1, in which the start codon of *Pten* was deleted in the genome to perform experiments (Fig. S1a). *Pten* deletion drove mouse ESCs toward a “domed” morphology (Fig. 1a), and *Pten*^*−/−*^ ESCs showed increased protein and mRNA levels of *Nanog*, *Oct4*, and *Klf4* (Fig. 1b and S1b). Immunostaining with anti-Nanog and anti-Oct4 antibodies confirmed that the expressions of Nanog and Oct4 proteins was upregulated in *Pten*^*−/−*^ ESCs (Fig. 1c). The elevated expression of pluripotency genes in *Pten*^*−/−*^ ESCs prompted us to investigate the colony formation ability of these cells. Alkaline phosphatase (AP) staining for *Pten*^*−/−*^ and WT ESCs was used to examine their colony morphology (Fig. 1d). Compared with WT ESCs, a greater proportion of *Pten*^*−/−*^ ESCs exhibited “domed” morphologies (green arrows) (Fig. 1d, e). The effect of *Pten* deletion on the maintenance of ESC pluripotency might vary in different *Pten*^*−/−*^ cell lines, thus we compared three independent *Pten*^*−/−*^ ESC lines with WT ESCs and found that all the *Pten*^*−/−*^ ESC lines consistently showed a more pronounced ground state and higher expression of pluripotency genes than the WT ESCs (Fig. S1c, d). In addition, re-expression of Pten in *Pten*^*−/−*^ ESCs by lentivirus system restored the expression of pluripotency genes (Fig. S1e). We performed transcriptomic analysis, and a pairwise comparison indicated that 242 genes were upregulated and 224 genes were downregulated in *Pten*^*−/−*^ ESCs (Fig. S1f). Multiple pluripotency markers were highly expressed in *Pten*^*−/−*^ ESCs (Fig. 1f). The principal component analysis (PCA) of gene expression revealed that *Pten*^*−/−*^ ESCs displayed distinct transcriptome profiles (Fig. S1g).

We also compared the expression of naïve and primed pluripotency genes between WT and *Pten*^*−/−*^ ESCs using heat map analysis and measured the mRNA levels of primed pluripotency genes (*Otx2*, *Pou3f1*, *Lefty1*, *Lin28a*, and *Dnmt3b*). *Pten*^*−/−*^ ESCs showed lower expression of the primed pluripotency genes and a higher expression of the naïve pluripotency markers (Fig. S2a, b). The protein level of the naïve marker Rex1 was also elevated in *Pten*^*−/−*^ ESCs (Fig. S2c). *Pten*^*−/−*^ ESCs exhibited an augmented proliferation ability (Fig. S2d), which is consistent with the previous results of Hong Wu showing that loss of Pten enhanced cellular self-renewal capacity^16,20^. Propidium iodide (PI) incorporation assay and cell apoptosis assay indicated that Pten deletion slightly suppressed apoptosis in ESCs (Fig. S2e, f). Taken together, these results indicated that *Pten* deletion could maintain naïve pluripotency of ESCs.

The ESCs were then induced to form EBs to further verify the effect of *Pten* deletion on ESC differentiation. As expected, there were higher levels of Nanog and Oct4 in *Pten*^*−/−*^ EBs than WT EBs, and the expression of Nanog and Oct4 was decreased after 3 days and 5 days, respectively, in WT and *Pten*^*−/−*^ EBs (Fig. S3a). The expression of Nanog was turned off completely at 7 days in WT EBs but persisted for 9 days in *Pten*^*−/−*^ EBs (Fig. S3a). In response to retinoic acid (RA), pluripotency gene expression is suppressed and ESCs undergo directed differentiation^21^. After treatment with RA for 3 days, the expression of Klf4 became undetectable in WT cells, while modest expression of Klf4 was retained in *Pten*^*−/−*^ cells, and the expression of Oct4 was higher in the *Pten*^*−/−*^ cells than WT cells (Fig. 1g). The mRNA levels of a series of differentiation markers decreased in *Pten*^*−/−*^ cells after RA treatment (Fig. S3b), and the protein levels of Mef2c (a mesoderm marker) and Gata4 (an endoderm marker) were reduced in *Pten*^*−/−*^ cells (Fig. S3c), thus *Pten* deletion might delay the early differentiation in mouse ESCs. *Pten*^*−/−*^ cells displayed greater proliferation ability than WT cells (Fig. S3d), and Pten deletion slightly suppressed cell apoptosis after RA treatment (Fig. S3e). It is consistent with the conclusion of a previous report that PTEN induces cell apoptosis^22^. Meanwhile, both WT and *Pten*^*−/−*^ ESCs could differentiate into mesodermal, ectodermal, and endodermal tissues (Fig. S3f).

We established protocols for ectoderm, mesoderm, and endoderm differentiation in vitro, and the expression of *Pax6* and *Sox2* (ectodermal markers) significantly increased in the *Pten*^*−/−*^ cells at day 7, while the expression of *Tubb3* (a neuron-specific marker) and *Krt8* (a skin marker) slightly decreased (Fig. S3g). The result is consistent with a previous conclusion that knockdown of Pten in the mouse brain has been shown to increase the proliferation of neural progenitor cells but not result in a bias of neuron differentiation^23^. However, the expression of mesodermal markers (*Mef2c*, *Kdr*, *MyoD1*, and *Mlc2v*) and endodermal markers (*Afp*, *Gata4*, and *FoxA2*) declined (Fig. S3g). We detected a dynamic change of protein levels of Pax6, Mef2c, and Gata4 during differentiation from day 0 to day 9. The protein level of Pax6 significantly increased from day 5, while the Mef2c and Gata4 levels were diminished (Fig. S3h). These results suggest that Pten deletion might delay the mesoderm and endoderm differentiation and that Pten regulates the early and later differentiation of ectodermal lineages through different mechanisms.

### *Pten* deletion sustains ESC pluripotency by blocking Gsk3β activity

**Fig. 2:**
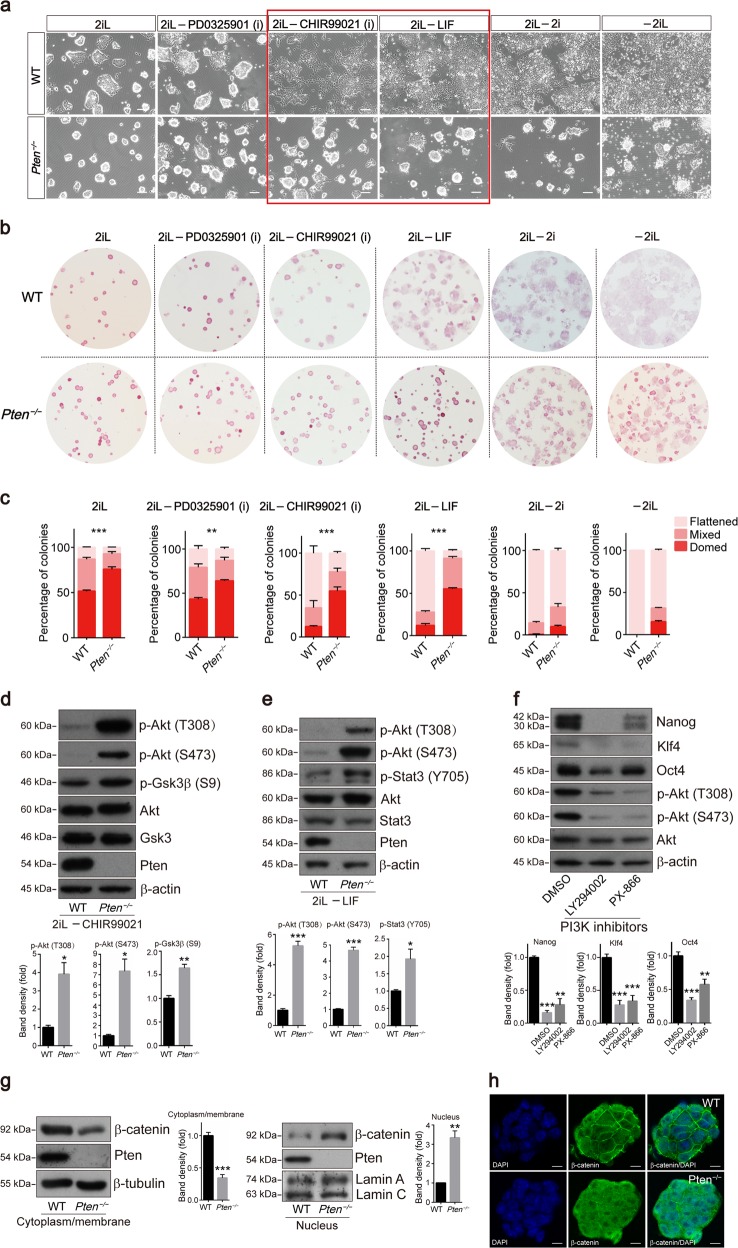
*Pten* deletion maintains ESCs by regulating the PI3K-Gsk3β signaling axis. **a** Phase-contrast images of WT and *Pten*^*−/−*^ ESCs cultured in medium including 2iL and in medium lacking PD0325901, CHIR99021, LIF, 2i, and 2iL, respectively. Scale bars, 100 µm. **b**, **c** Analysis of AP staining of WT and *Pten*^*−/−*^ ESC colonies cultured in medium including 2iL and in medium lacking PD0325901, CHIR99021, LIF, 2i, and 2iL, respectively. Without CHIR99021 and LIF, respectively, *Pten*^*−/−*^ ESC colonies displayed more than 50% domed morphology, but WT ESC colonies showed a large reduction in domed morphology. Error bars indicate mean ± SEM (*n* = 3), and 80 colonies were scored in each replicate. **d** Western blot analysis of WT and *Pten*^*−/−*^ ESCs cultured in medium without CHIR99021 showing the phosphorylation of Akt at T308 and S473 and the phosphorylation of Gsk3β at S9. **e** Western blot analysis of WT and *Pten*^*−/−*^ ESCs cultured in medium without LIF showing the phosphorylation of Akt at T308 and S473 and the phosphorylation of Stat3 at Y705. **f** Western blot analysis of *Pten*^*−/−*^ ESCs treated with PI3K inhibitors LY294002 and PX-866 showing the expression of the pluripotency markers and the phosphorylation of Akt at T308 and S473. **g** Protein levels of β-catenin in the cytoplasm/membrane and nucleus of WT and *Pten*^*−/−*^ ESCs. **h** Immunofluorescence staining for β-catenin in WT and *Pten*^*−/−*^ ESCs. DNA was stained with DAPI to indicate nuclei. Scale bars, 10 µm.

ESCs established in the presence of 2iL are postulated to represent the ground state pluripotency^24^. Akt inhibits Gsk3β activity through the phosphorylation of Ser9^25,26^, and a previous report mentioned that PTEN knockdown in human ESCs results in augmented self-renewal, survival, and proliferation but has no effects on GSK3 activity^15^. In our results, WT and *Pten*^*−/−*^ ESCs were cultured in medium including 2iL and in medium lacking PD0325901, CHIR99021, LIF, 2i, and 2iL, respectively (Fig. 2a). We found that *Pten*^*−/−*^ ESCs still displayed a “domed” morphology in culture medium lacking CHIR99021 or LIF, while a large proportion of WT ESCs displayed a “flattened” morphology (Fig. 2a–c). The activity of the PI3K-Gsk3β pathway was examined when ESCs were cultured in medium without CHIR99021. The phosphorylation of Akt significantly increased in *Pten*^*−/−*^ ESCs, and the Gsk3β phosphorylation also increased, which is supposedly correlated with the inactivation of Gsk3β (Fig. 2d, S4d, and S4g). Besides, the protein expression levels of Nanog, Klf4, and Oct4 were increased in *Pten*^*−/−*^ ESCs in the absence of CHIR99021 (Fig. S4c). Sox2 is a substrate of Akt, and we measured the total level and phosphorylation level of Sox2 in ESCs and EBs. Intriguingly, loss of Pten suppressed the expression of Sox2 in ESCs (Fig. S4e) but promoted the expression in EBs (Fig. S4f). In addition, the phosphorylation levels of Sox2 increased both in *Pten*^*−/−*^ ESCs and EBs when the phosphorylation levels were normalized to the total level of Sox2 (Fig. S4e, f). This indicated that loss of Pten activates Akt and that there might be a complex mechanism, through which Pten regulates the expression of Sox2 at different stages during ESCs differentiation.

Mouse ESCs are maintained by using the cytokine LIF to activate Stat3 signaling^9–11^, and PTEN negatively regulates PI3K/mammalian target of rapamycin (mTOR)^27^, which promotes STAT3 activity^28^. After the removal of LIF, the ESC maintenance by *Pten* deletion might be due to the activation of Stat3 (Fig. 2e). AKT lies at a signaling node downstream of PI3K, and the PI3K/AKT signaling pathway is sufficient to maintain the self-renewal and survival of stem cells^29^. We evaluated the effects of PI3K inhibitors (LY294002 and PX-866) on the pluripotency of *Pten*^*−/−*^ ESCs and found that PI3K inhibitors significantly abated the expression of pluripotency genes by inhibiting Akt activity (Fig. 2f). A previous result showed that PTEN deficiency augmented the levels of p-S6 for activating the AKT/mTOR signaling pathway in human ESCs^15^. To identify alternative mechanisms involved in Pten-regulated ESC maintenance, we assessed the activity of several signaling pathways, including the MAPK and mTOR pathways, and loss of Pten promoted the mTOR signaling pathway (Fig. S5d). PI3K/Akt/mTOR is known to play key roles in cell adhesion, proliferation, and survival. In the PI3K/Akt/mTOR pathway, Pten acts as a brake upstream of Akt, and Akt activates mTOR via phosphorylation of TSC2, and this regulation mechanism was revealed in many biological processes^30,31^. mTOR inhibition by rapamycin could rescue the expression of Klf4 and Nanog in *Pten*^*−/−*^ ESCs (Fig. S5e). Our results indicate that PI3K/Akt/mTOR might be another signaling pathway regulated via Pten in modulating ESCs’ naïve pluripotency.

β-catenin is a downstream factor of Gsk3β, and Wnt/β-catenin signaling plays important roles in ESC self-renewal^32,33^. In our results, the cytoplasm/membrane distribution of β-catenin was reduced in *Pten*^*−/−*^ ESCs, while a higher nuclear abundance of β-catenin was observed (Fig. 2g). The immunofluorescence result showed that β-catenin was enriched in the membrane of WT ESCs (Fig. 2h). We also verified that the mRNA levels of Wnt-target genes such as *c-Myc*, *Jun*, *Dkk1*, *Axin2*, and *Sp5* increased (Fig. S4i) and that the protein levels of c-Myc and Jun also increased in *Pten*^*−/−*^ ESCs (Fig. S4j). We supposed that inhibition of Gsk3β in *Pten*^*−/−*^ ESCs might stabilize β-catenin and that the stabilized β-catenin translocated to the nucleus to mimic stimulation of canonical Wnt signaling, which restricts the conversion from naïve to primed pluripotency^34^.

ESCs were cultured in medium with different concentrations of CHIR99021 or LIF (Fig. S4a and S5a). CHIR99021 is utilized to maintain naïve pluripotency in mouse ESCs^35^. An increased proportion of WT ESCs exhibited “domed” morphology as the concentration of CHIR99021 increased, and the proportion of ground-state WT ESCs was equivalent to *Pten*^*−/−*^ ESCs at 4 µM and 5 µM CHIR99021 (Fig. S4a, b). The protein level of pluripotency markers in WT ESCs was also similar to *Pten*^*−/−*^ ESCs at 4 µM and 5 µM CHIR99021 (Fig. S4h). These results indicated that *Pten*^*−/−*^ ESCs might sustain ground pluripotency with lower concentrations of CHIR99021 than those needed by WT ESCs, supporting the hypothesis that Gsk3β kinase is more inhibited in *Pten*^*−/−*^ ESCs. It is consistent with the previous data that *Pten*^*−/−*^ ESCs showed higher phosphorylation levels of Gsk3β which is supposedly correlated with the inactivation of Gsk3β (Fig. 2d). In addition, LIF could not revert WT ESCs to a similar proportion of domed colonies as *Pten*^*−/−*^ ESCs (Fig. S5a, b). Fig. S5c showed that the loss of Pten had no effect on the activity of Stat3 when ESCs were cultured in medium with 2iL. This suggests that LIF/Stat3 signaling might be not the main signaling pathway regulated by Pten in modulating ESC pluripotency.

These results indicated that *Pten*^*−/−*^ ESCs maintain pluripotency primarily through inhibiting Gsk3β, similar to the role of the Gsk3β-inhibitor CHIR99021.

### The Pten-inhibitor SF1670 contributes to ESC maintenance

**Fig. 3:**
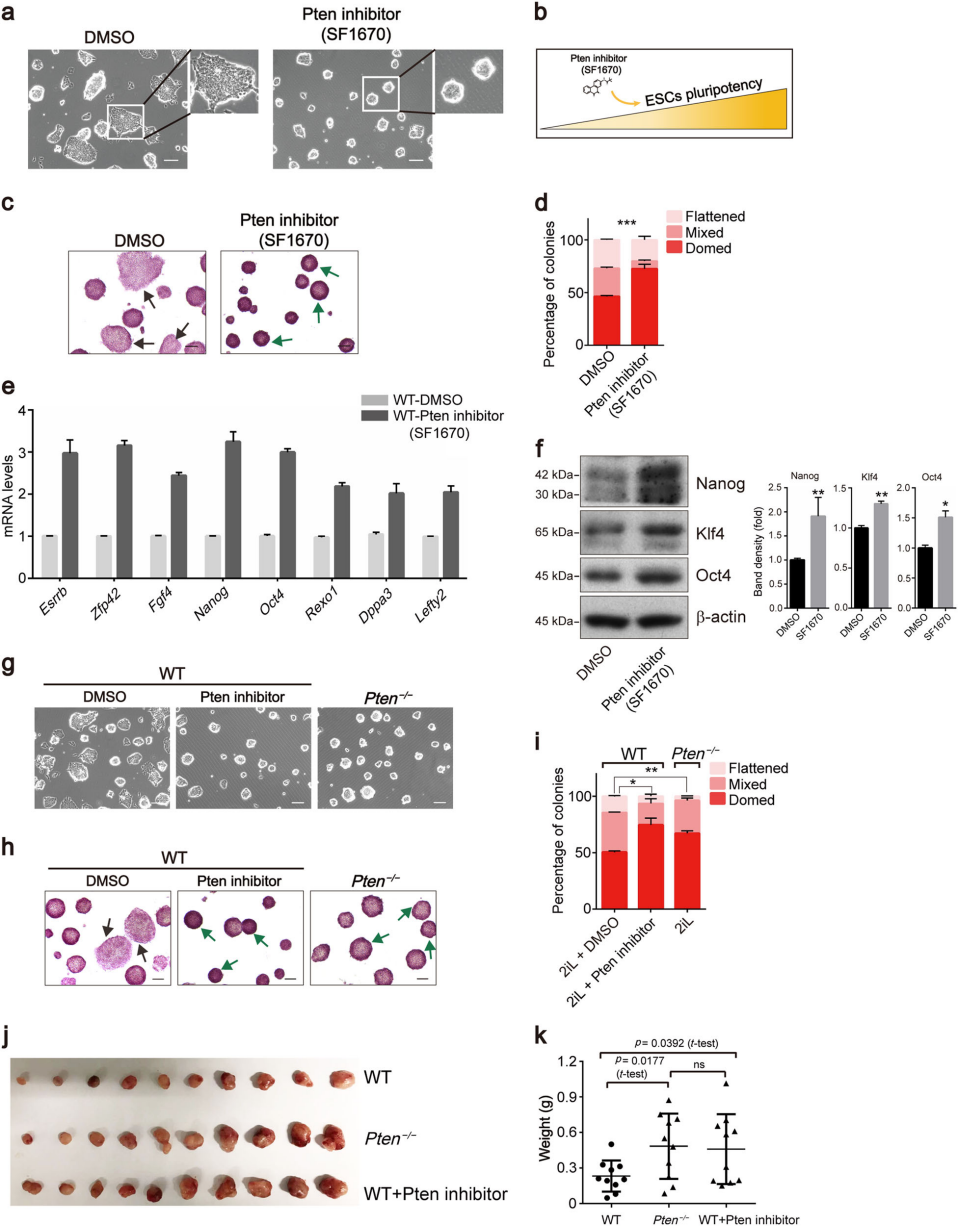
The Pten-inhibitor SF1670 contributes to sustaining ESC pluripotency. **a** Phase-contrast images of WT ESCs treated with DMSO and the Pten-inhibitor SF1670 when cultured in medium with 2i and LIF (2iL). Scale bars, 100 µm. **b** Pten-inhibitor SF1670 promotes the ESC pluripotency. **c** Alkaline phosphatase staining of WT ESC colonies treated with DMSO and SF1670. There are more flattened colonies (black arrow) in DMSO-treated ESCs, and more domed colonies (green arrow) in SF1670-treated ESCs. **d** Analysis of colony morphology of WT ESCs treated with DMSO and SF1670. Error bars indicate mean ± SEM (*n* = 3), and 80 colonies were scored in each replicate. **e** qRT-PCR analysis of mRNA expression of pluripotency markers (*Esrrb*, *Zfp42*, *Fgf4*, *Nanog*, *Oct4*, *Rexo1*, *Dppa3*, and *Lefty2*) in DMSO-treated ESCs and SF1670-treated ESCs. Error bars indicate mean ± SD (*n* = 3). **f** Western blot analysis of WT ESCs treated with DMSO and SF1670 showing the expression of the pluripotency markers Nanog, Klf4, and Oct4. **g** Phase-contrast images of DMSO-treated WT, SF1670-treated WT, and *Pten*^*−/−*^ ESCs when cultured in medium with 2iL. Scale bars, 100 µm. **h** AP staining of DMSO-treated WT, SF1670-treated WT, and *Pten*^*−/−*^ ESC colonies when cultured in medium with 2iL. **i** Analysis of colony morphology of DMSO-treated WT, SF1670-treated WT, and *Pten*^*−/−*^ ESC colonies. Error bars indicate mean ± SEM (*n* = 3), and 80 colonies were scored in each replicate. **j** Nude mice were injected with WT ESCs, *Pten*^*−/−*^ ESCs, and SF1670-treated WT ESCs on the left and right sides, respectively. Teratomas were generated at the contralateral positions aligned at the same positions in each treatment. **k** Weights (g) of teratomas from WT ESCs, *Pten*^*−/−*^ ESCs, and SF1670-treated WT ESCs.

PI3K/Akt signaling is crucial for stem cell self-renewal, and the active form of Akt sustains pluripotency in mouse and primate ESCs^36,37^. We hypothesized that the activation of the PI3K/Akt pathway by small-molecule inhibitors of Pten maintains the pluripotency of ESCs. We treated mouse ESCs with several Pten inhibitors (data not shown) and found that the Pten-specific inhibitor SF1670 maintained the pluripotency of ESCs (Figs. 3a, b, and S6a). SF1670 maintained ESC pluripotency as indicated by the increased proportion of colonies with a “domed” morphology (green arrows), while DMSO-treated ESCs showed a higher proportion of colonies with a “flattened” morphology (black arrows) (Fig. 3c, d). The transcript levels of several pluripotency genes were significantly increased by SF1670 (Fig. 3e). The protein levels of pluripotency genes were also elevated in ESCs treated with SF1670 (Fig. 3f and S6e). SF1670 is a specific inhibitor of Pten, and as expected, the SF1670-treated WT ESCs exhibited similar “domed” morphologies as the *Pten*^*−/−*^ ESCs (Fig. 3g, h, i). We also evaluated the teratoma formation ability and found that the weight of teratomas derived from both SF1670-treated WT ESCs and *Pten*^*−/−*^ ESCs increased compared with those derived from WT ESCs (Fig. 3j, k). Fig. S6b shows the colony morphology of WT ESCs, WT ESCs treated with different concentrations of SF1670, and *Pten*^*−/−*^ ESCs cultured in ESC culture medium. The proportion of cells with “domed” morphologies rose as the SF1670 concentration increased (Fig. S6c, d) indicating that the Pten-inhibitor SF1670 contributed to ESC maintenance.

### The Pten-inhibitor SF1670 plays a similar role to the Gsk3β-inhibitor CHIR99021 in ESC maintenance

**Fig. 4:**
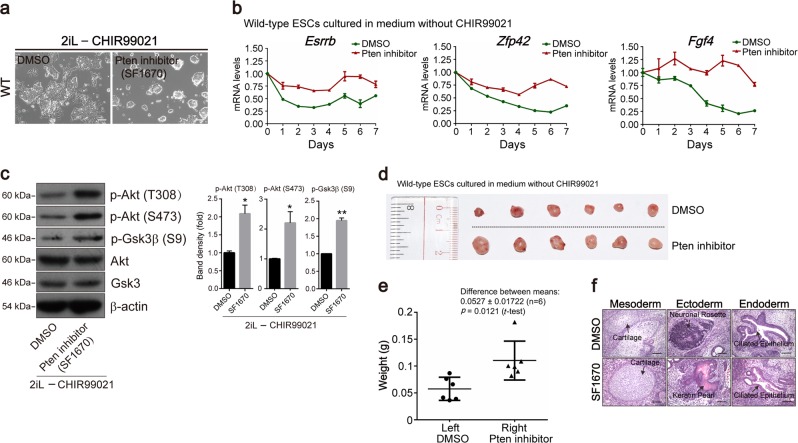
Pten-inhibitor SF1670 plays a similar role to CHIR99021 in maintaining the pluripotency of ESCs. **a** Phase-contrast images of DMSO-treated and SF1670-treated WT ESCs cultured in medium without CHIR99021. Scale bars, 100 µm. **b** Q-PCR analysis of pluripotency marker (*Esrrb*, *Zfp42*, and *Fgf4*) expression in DMSO-treated and SF1670-treated ESCs cultured in medium without CHIR99021 from day 0 to day 7. Error bars indicate mean ± SD (*n* = 3). **c** Western blot analysis of WT ESCs treated with DMSO and SF1670 in medium without CHIR99021 showing the phosphorylation of Akt at T308 and S473 and the phosphorylation of Gsk3β at S9. **d** Nude mice were injected with DMSO-treated and SF1670-treated WT ESCs on the left and right sides, respectively. Teratomas were generated at the contralateral positions aligned at the same positions in each treatment. **e** Weights (g) of teratomas from DMSO-treated and SF1670-treated ESCs. **f** DMSO-treated and SF1670-treated ESCs were injected into immunodeficient mice and produced teratocarcinomas containing tissues representative of the three germ layers (mesoderm, ectoderm, and endoderm).

SF1670 is a small-molecule inhibitor of Pten and can increase the PIP_3_ level in transfused neutrophils^38^. The DMSO-treated and SF1670-treated WT ESCs were cultured in medium including 2iL and in medium lacking PD0325901, CHIR99021, LIF, 2i, and 2iL, respectively, and SF1670 sustained the “domed” morphology of WT ESCs in the absence of CHIR99021 (Fig. S7a, b), while LIF could not be substituted by SF1670 for inadequate activation of Stat3 (Fig. S7a, b, e). SF1670 at 2 µM also eliminated the loss of the “domed” morphology of ESCs seen in the absence of CHIR99021 (Figs. 4a, and S7c). We also measured the transcript levels of pluripotency genes (*Esrrb*, *Zfp42*, and *Fgf4*) in WT ESCs at different time points in the absence of CHIR99021. Their expression levels were higher in SF1670-treated ESCs than in DMSO-treated ESCs (Fig. 4b). Immunostaining result confirmed that the expression of Nanog protein was upregulated in SF1670-treated ESCs (Fig. S7d). The phosphorylation levels of Akt at T308 and S473 and Gsk3β at S9 were significantly augmented in SF1670-treated ESCs cultured in medium without CHIR99021 (Fig. 4c). The protein level of the naïve marker Rex1 was slightly elevated in SF1670-treated ESCs (Fig. S7f), and the proportion of Rex1 positive cells was also slightly increased in SF1670-treated ESCs compared to DMSO-treated ESCs (Fig. S7g). In addition, the in vivo teratoma formation ability was examined, and the teratomas derived from SF1670-treated ESCs were larger than those formed from DMSO-treated ESCs (Fig. 4d, e). Meanwhile, the DMSO-treated and SF1670-treated ESCs could differentiate into mesodermal, ectodermal, and endodermal tissues (Fig. 4f). On the other hand, the cell proliferation ability of SF1670-treated ESCs increased compared with DMSO-treated ESCs (Fig. S7h), and SF1670 slightly suppressed the apoptosis of ESCs (Fig. S7i).

These results indicate that Pten inhibition by SF1670 plays a similar role to the Gsk3β-inhibitor CHIR99021 in ESC maintenance.

### Pten activation by S380A, T382A, and T383A (Pten-A3) mutations suppresses ESC pluripotency

**Fig. 5:**
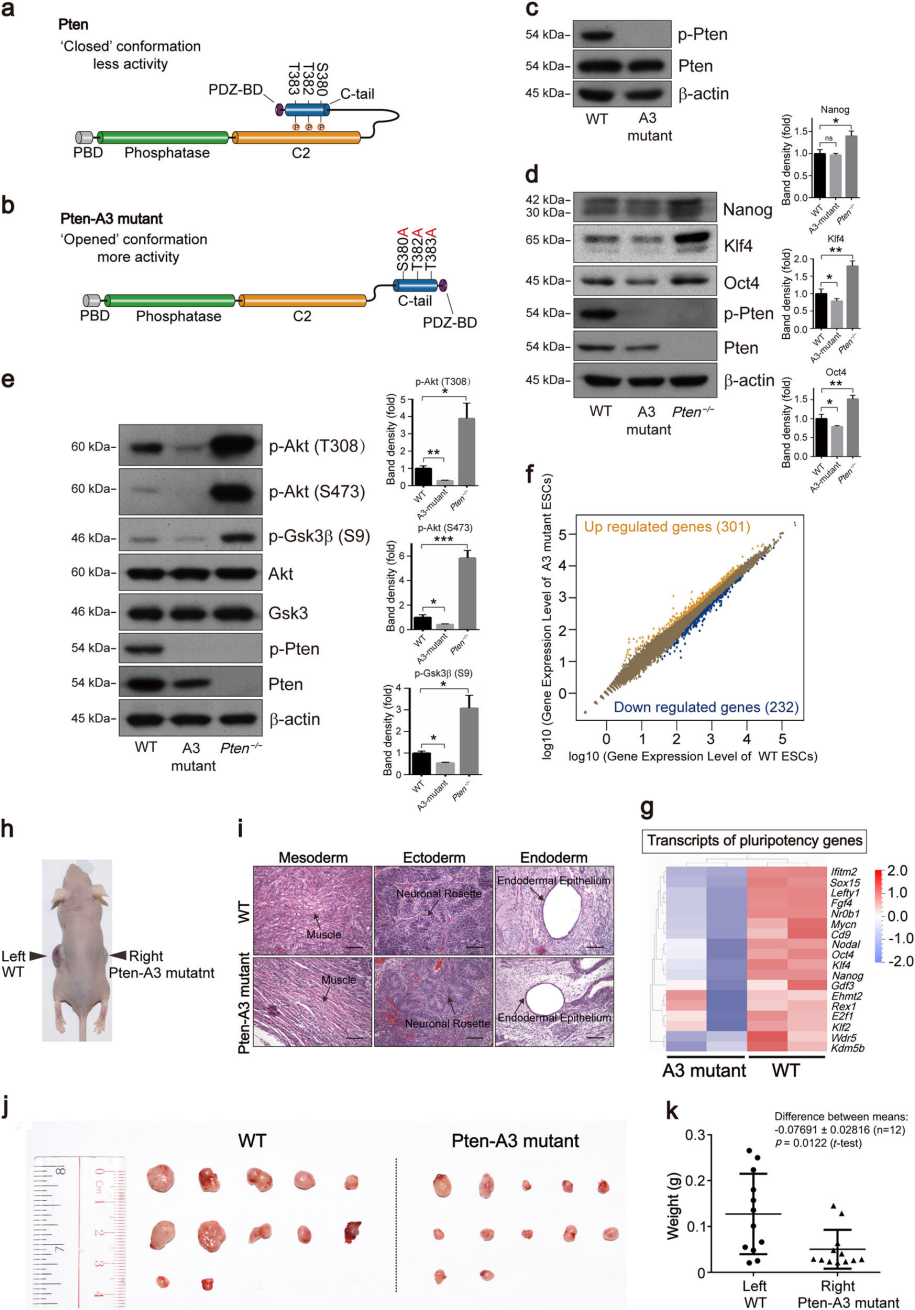
Pten activation by S380A, T382A, T383A mutations suppresses ESC pluripotency. **a**, **b** The domain structure of Pten in the “closed” conformation and “opened” conformation. **c** Western blots analysis showing the loss of phosphorylation in the Pten-A3 mutant ESCs. **d** Western blot analysis of WT, Pten-A3 mutant, and *Pten*^*−/−*^ ESCs showing the expression of the pluripotency markers Nanog, Klf4, and Oct4. **e** Phosphorylation of Akt at S473 and T308 and phosphorylation of Gsk3β at S9 by Western blot. **f** Scatter plots of transcript expression in WT and Pten-A3 mutant ESCs. Expression values are shown on a log10 scale. Yellow dots indicate upregulated genes in *Pten*^*−/−*^ ESCs, and blue dots indicate downregulated genes (fold-change cutoff = 1.5, FDR threshold ≤ 0.05). **g** Heat map of FPKM values of pluripotency genes in WT and Pten-A3 mutant ESCs. The heat map was normalized with sigma-normalization per row. See also Table S3. **h** A nude mouse injected with WT and Pten-A3 mutant ESCs on the left and right sides, respectively. **i** WT and Pten-A3 mutant ESCs were injected into immunodeficient mice and produced teratocarcinomas containing tissues representative of the three germ layers (mesoderm, ectoderm, and endoderm). **j** Teratomas from WT and Pten-A3 mutant cells generated at the contralateral positions aligned at the same positions in each genotype. **k** Weights (g) of teratomas from WT and Pten-A3 mutant cells.

Phosphorylation of the C-terminal of PTEN at S380, T382, and T383 can inhibit its phosphatase activity and favor its stabilization^39,40^. We have shown that Pten inhibition or deletion sustained the ESC pluripotency (Figs. 1–4). To explore the underlying molecular mechanism, we hypothesized that the pluripotency of ESCs would be influenced when Pten was activated by S380A, T382A, and T383A (Pten-A3) mutations that prevent their phosphorylation (Fig. 5a, b). Pten-A3 mutant ESCs were generated by using the CRISPR-Cas9 system (Fig. S8a). Phosphorylation of S380, T382, and T383 could not be detected in Pten-A3 mutant ESCs by Western blot (Fig. 5c and S8b). The protein levels of Oct4 and Klf4 were decreased in Pten-A3 mutant ESCs but were significantly increased in *Pten*^*−/−*^ ESCs (Fig. 5d). We also measured the expression of pluripotency genes in two other Pten-A3 mutant cell lines, and the result was consistent with Fig. 5d (Fig. S8b). Overexpression of Pten in Pten-A3 mutant ESCs could restore the expression of pluripotency genes (Fig. S8c). We stained the ESC colonies with an anti-Nanog antibody, and the signal in the Pten-A3 mutant ESCs was weaker than in the WT ESCs (Fig. S8d). Furthermore, AP staining showed that the proportion of domed colonies was decreased in the Pten-A3 mutant ESCs (Fig. S8e, f, g), and the proliferation ability of Pten-A3 mutant ESCs was also inhibited (Fig. S8h).

The experiments described above showed that the PI3K-Gsk3β pathway is involved in regulating the ESC pluripotent state via Pten. The phosphorylation of Akt and Gsk3β was significantly decreased in the Pten-A3 mutant ESCs compared with WT ESCs but was significantly increased in the *Pten*^*−/−*^ ESCs (Fig. 5e). The stability and activity of Pten are linked to the phosphorylation of the S380, T382, and T383 residues in the C-terminal tail^41^. Pten activity was inhibited, but the expression of Pten also decreased in the Pten-A3 mutant ESCs (Fig. 5d, e). Thus, there was a balance for Pten-3A mutant ESCs in the regulation of ESC pluripotency between inhibition of Pten activity and the degradation of Pten.

Transcriptome analysis of WT and Pten-A3 mutant ESCs was performed, and a scatter plot analysis showed the difference between the WT and Pten-A3 mutant ESCs (Fig. 5f). The reduced expression of pluripotency markers in Pten-A3 mutant ESCs was shown in the heat map (Fig. 5g). We then examined the teratoma formation ability of Pten-A3 mutant ESCs in vivo. The WT and Pten-A3 mutant ESCs were injected into immunodeficient mice and both gave rise to multidifferentiated teratomas that contained mesodermal, ectodermal, and endodermal tissues (Fig. 5h, i), while the teratomas derived from Pten-A3 mutant cells were significantly smaller than those derived from WT ESCs (Fig. 5j, k).

Our results indicate that phosphorylation levels of Pten at S380, T382, and T383 are important for Pten to regulate the ESC pluripotent state by inhibiting the activity of Pten and promoting Akt activity.

### Suppression of *Pten* regulates lineage choice and differentiation during embryoid body formation

**Fig. 6:**
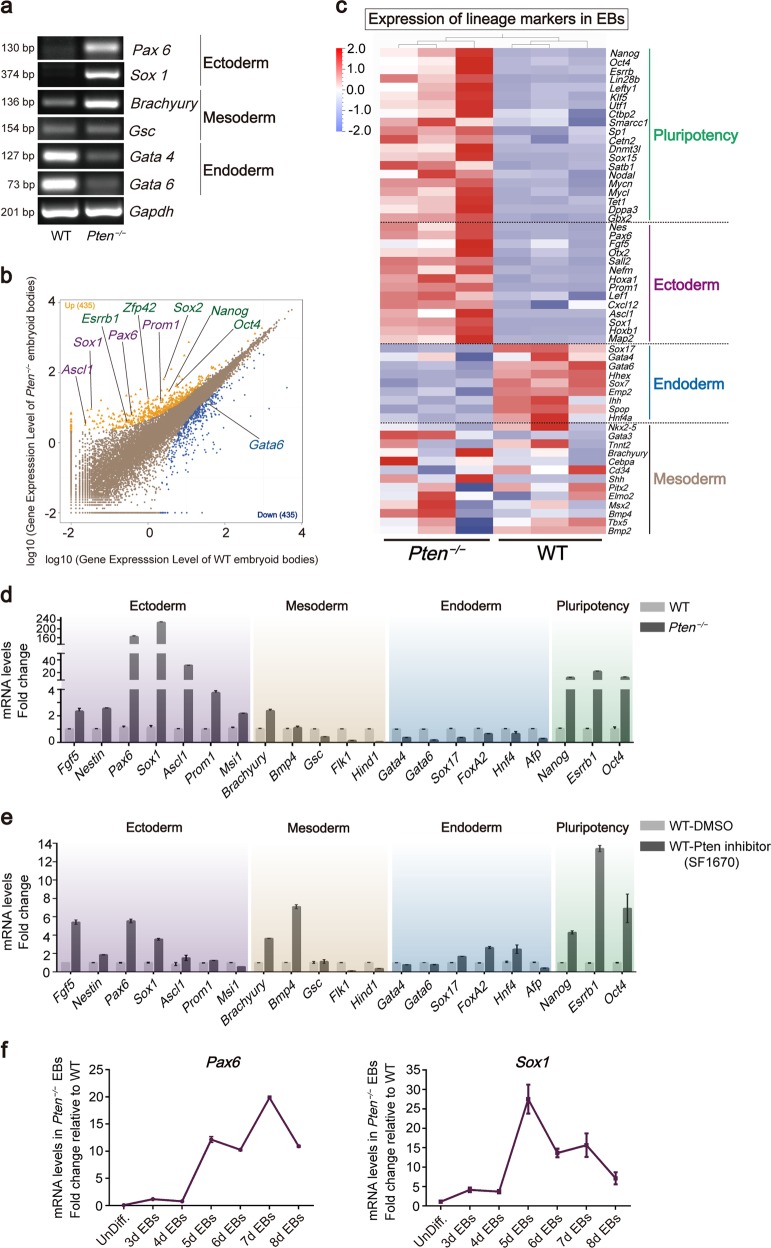
*Pten* deletion and Pten inhibition regulate lineage choice and differentiation in mouse EBs. **a** RT-PCR analysis of EBs derived from WT and *Pten*^*−/−*^ ESCs showing the expression of lineage-specific markers. **b** Scatter plot of transcript expression in WT and *Pten*^*−/−*^ EBs. Expression values are shown on a log10 scale. Yellow dots indicate upregulated genes in *Pten*^*−/−*^ EBs, and blue dots indicate downregulated genes. Probability ≥ 0.8 and abs (log2(Y/X)) ≥ 1. **c** Heat map of FPKM values of pluripotency, ectoderm, endoderm, and mesoderm markers in WT and *Pten*^*−/−*^ EBs. The heat map was normalized with sigma-normalization per row. See also Table S4. **d** The expression of lineage-specific markers was assessed by qRT-PCR in WT and *Pten*^*−/−*^ EBs. Error bars indicate mean ± SD (*n* = 3). **e** The expression of lineage-specific markers was assessed by qRT-PCR in WT EBs treated with DMSO or SF1670. Error bars indicate mean ± SD (*n* = 3). **f** Expression of ectoderm markers *Pax6* and *Sox1* in WT and *Pten*^*−/−*^ EBs at different days. Error bars indicate mean ± SD (*n* = 3).

Pten deletion or inhibition sustained ESC pluripotency, suggesting that *Pten* plays an important role during early embryogenesis. We measured the expression of the three embryonic germ layer markers by PCR. The expression of the ectoderm markers was significantly upregulated in *Pten*^*−/−*^ EBs and the endoderm markers were downregulated (Fig. 6a).

We used RNA-Seq to compare the transcriptomes of EBs derived from *Pten*^*−/−*^ and WT ESCs. A pairwise comparison indicated that 435 genes were significantly upregulated in *Pten*^*−/−*^ EBs, including ectoderm genes (*Ascl1*, *Sox1*, *Pax6*, *and Prom1*) and pluripotency genes (*Nanog*, *Oct4*, *Esrrb1*, *Sox2, and Zfp42*), and 435 genes were significantly downregulated, including the endoderm gene *Gata6* (Fig. 6b). PCA and Pearson correlation coefficients for all gene expression showed distinct expression patterns in WT and *Pten*^*−/−*^ EBs (Fig. S9a, b). The expression of the lineage-specific markers in WT and *Pten*^*−/−*^ EBs was visualized in the heat map, and ectoderm and pluripotency markers were increased in *Pten*^*−/−*^ EBs while endoderm markers were decreased (Fig. 6c). We measured the expression of the representative lineage-specific markers by qRT-PCR, and the expressions of the ectoderm and pluripotency markers were elevated in *Pten*^*−/−*^ EBs compared with WT EBs, but endoderm markers were decreased (Fig. 6d). In addition, the expression patterns of the ectoderm and pluripotency markers in WT EBs treated with 2 µM SF1670 were consistent with the results in *Pten*^*−/−*^ EBs (Fig. 6d, e). The expression levels of ectoderm markers *Pax6* and *Sox1* were measured on different days, and there was a significant difference between WT and *Pten*^*−/−*^ EBs after culturing for 8 days (Fig. 6f). Fig. S9c shows the expression of ectoderm markers (*Sox1*, *Sox3*, *Brn2*, *Ascl1*, *Nestin*, *Msi1*, *Prom1*, and *Pax6*) for three WT and three *Pten*^*−/−*^ EBs.

These results suggest that *Pten* deletion or Pten inhibition promotes ectoderm differentiation, inhibits endoderm differentiation, and maintains the pluripotency of mouse ESCs.

## Discussion

**Fig. 7:**
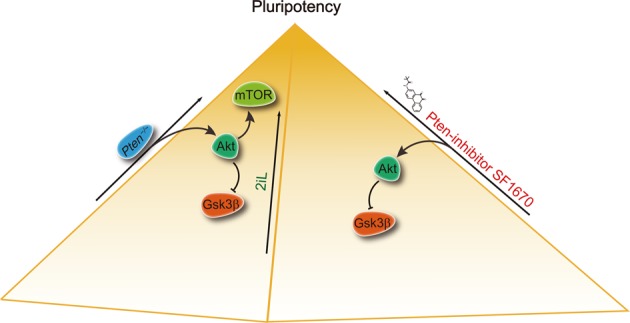
A model for how *Pten* deletion or inhibition promotes ESC pluripotency. *Pten* deletion or inhibition promotes naïve pluripotency of mouse ESCs by activating Akt and further suppressing Gsk3β. In addition, the Pten/Akt/mTOR signaling pathway is also involved in regulating ESC maintenance.

Mouse and human ESC lines are derived from blastocysts and provide important models to investigate early embryogenesis^1,2^. ESCs can be maintained by some small-molecule inhibitors such as Gsk3β-specific inhibitors and MAPK inhibitors. Here, our results showed that *Pten* deletion and the Pten-inhibitor SF1670 sustained ESC pluripotency by inhibiting Gsk3β (Fig. 7). This study thus demonstrates the functions of Pten in modulating the naïve pluripotency of ESCs and differentiation and has identified the regulatory mechanism.

Inhibition of Pten and p53 can drive increased Myc protein levels and promote an undifferentiated state with high renewal potential in mouse neural stem cells^13^. This is also an indication that *Pten* can regulate stem cell differentiation. We calculated the proportion of suspended and attached EBs of WT and *Pten*^*−/−*^ EBs. Intriguingly, fewer of *Pten*^*−/−*^ EBs were attached on the Petri dishes (Fig. S3i). Lineage-specific differentiation is influenced by pluripotency states, for example, naïve versus primed states of pluripotent stem cells are not functionally equipotent in lineage-specific differentiation, and naïve pluripotent stem cells possess impaired mesoderm and endoderm capacity but enhance the capacity for neural differentiation^42^. In our results, Pten ablation promoted mouse ESCs to a more naïve pluripotency (Fig. S2a, b), and *Pten*^*−/−*^ ESCs displayed an augmented differentiation ability for neural progenitor cells and delayed the mesoderm and endoderm differentiation (Fig. S3g, h). The effects of Pten deletion on lineage differentiation might be due to the naïve state.

Gene Ontology analysis by using DAVID functional innovation was performed with the RNA-seq data of WT and *Pten*^*−/−*^ EBs, WT and *Pten*^*−/−*^ ESCs, and WT and Pten-A3 mutant ESCs and showed that the PI3K/Akt pathway was enriched in these cell lines (Fig. S9d, e, f). Different signaling pathways were enriched in various cell lines, for example, Ras signaling pathway and Rap1 signaling pathway were enriched in wild-type and *Pten*^*−/−*^ EBs (Fig. S9d), and Hippo signaling pathway, Calcium signaling pathway, and cAMP signaling pathway were enriched in wild-type and Pten-A3 mutant ESCs (Fig. S9f). The enrichment of signaling pathways in different cell lines suggests that there may exist a complex and dynamic signaling network at different stages of embryonic development. According to our current results, we supposed that Pten modulated ESC pluripotency primarily through regulating PI3K/Akt/Gsk3β signaling pathway.

Our data showed that inhibition of Pten maintained ESCs pluripotency, and we defined the underlying molecular mechanism. This supports previous work showing that inhibition of Pten promotes the generation of induced pluripotent stem cells from mouse embryonic fibroblasts^43^. We hypothesize that Pten inhibition is potentially useful in mouse ESC culture and regenerative medicine because of its role in ESC maintenance.

## Materials and methods

### Animals

All mouse procedures were performed in accordance with the recommendations of the Animal Experimentation Ethics Committee of The Chinese University of Hong Kong. The protocol was approved by the Animal Experimentation Ethics Committee of the Chinese University of Hong Kong (Ref No. 17–206-MIS).

### Mouse ESC culture

The mouse E14 cell line was purchased from ATCC (ES-D3, ATCC, CRL-1934). Mouse ESCs were cultured in DMEM/F12 containing GlutaMAX and sodium pyruvate (Life Technologies, 10565) with 15% fetal bovine serum (Hyclone, SH30071), nonessential amino acid solution (Life Technologies, 11140), and β-mercaptoethanol (Life Technologies, 31350) with 1 × 10^3^ units/ml LIF (Millipore, ESG1106), 1 µM PD0325901 (Sigma, PZ0162), and 2.5 µM CHIR99021 (Sigma, SML1046). The cell line was tested for mycoplasma contamination.

### *Pten* deletion and Pten-A3 mutations in mouse ESCs by CRISPR-Cas9 technologies

Guide sequences of sgRNA were incorporated into the pSpCas9(BB)-2A-GFP vector (Addgene, 48138) containing *Cas9* and green fluorescent protein (*GFP*) genes. ESCs were transfected with constructs and then subjected to cell sorting of GFP-positive cells after transfection for 48 h (Figs. S1a and S8a). Finally, the *Pten*^*−/−*^ and Pten-A3 mutant cell lines were identified by sequencing, and the cells were stored in liquid nitrogen. The sgRNA sequence and sequences of the primers are shown in Table S1.

### Colony-formation assay

For the colony formation assay, ESCs were cultured in gelatin-coated plates at 250 cells per cm^2^ for 4 days. AP activity was detected by using an AP Staining Kit II according to the product manual (STEMGENT, 00–0055). Sample size was determined based on previous experience. At least three times of biological replicates have been performed for each cell line, and 80 colonies were scored in each replicate. The person collecting outcome measures and analyzing data was blinded to group allocation.

### Teratoma formation and histological analysis

ESCs were dissociated and suspended in phosphate-buffered saline (PBS) supplemented with 50% Matrigel (BD, 354234) at 1 × 10^6^ cells/ml. A total of 5 × 10^4^ cells were injected subcutaneously into recipient nude mice. To minimize the inter-individual variability in teratoma growth, two groups of ESCs were injected in the left and right side, respectively, in the same individuals. Sample size was determined based on previous experience and chosen based on putative effects sizes in pilot experiments. Animals were allocated to injection groups with different cell lines by randomization, and the person performing animal injection was blinded the sample groups. Cells (lines WT ESCs, *Pten*^*−/−*^ ESCs, and SF1670-treated WT ESCs) were injected into nude mice (ten mice per line); cells (lines DMSO-treated WT ESCs and SF1670-treated WT ESCs) were injected into nude mice (six mice per line); and cells (lines WT ESCs and Pten-A3 mutant ESCs) were injected into nude mice (12 mice per line). Three weeks later, the teratomas were isolated, fixed with 4% paraformaldehyde (PFA) in PBS, and stained with hematoxylin and eosin.

### Immunofluorescence

After culturing for 3 days, ESCs were fixed with 4% PFA for 20 min and incubated in PBS with 0.3% Triton-X 100 for 10 min. After blocking with 5% bovine serum albumin in PBS, ESCs were stained with primary antibodies and incubated overnight at 4 °C. The next day, samples were incubated with donkey anti-rabbit IgG highly cross-adsorbed secondary antibodies (Life Technologies, A16036) for 2 h at RT. The following antibodies were used for immunofluorescence analysis: anti-Nanog (Cell Signaling Technology, 8822), anti-β-catenin (Cell Signaling Technology, 8480), and anti-Oct4 (Cell Signaling Technology, 2840).

### Western blot

Cells were scraped from culture plates and incubated for 30 min in ice-cold lysis buffer containing protease inhibitor cocktails. For nuclear and cytoplasmic fractionation, the cells were collected by trypsin digestion and incubated in the cytoplasmic lysis buffer (10 mM Hepes, 1.5 mM MgCl_2_, 10 mM KCl, 0.5 mM DTT, 300 mM sucrose, 0.1% NP-40, and protease inhibitors cocktail). Cells were lysed for 10 min on ice and spun for 15 s at 12,000 rpm to collect the cytosolic lysate. Pellets were washed two times with cytoplasmic lysis buffer and lysed with nuclear lysis buffer (50 mM Hepes, 250 mM KCl, 0.1 mM EDTA, 0.1% NP-40, 0.1% glycerol, protease inhibitors cocktail) for 30 min on ice. The lysates were spun for 20 min at 14,000 rpm to collect nuclear lysates. The following antibodies were used for western blot analysis: anti-Nanog (Cell Signaling Technology, 8822), anti-Oct4 (Cell Signaling Technology, 2840), anti-Klf4 (Cell Signaling Technology, 4038), anti-Pten (Cell Signaling Technology, 9188), anti-β-actin (Immunoway, YM3028), anti-p-T308-Akt (Cell Signaling Technology, 13038), anti-p-S473-Akt (Cell Signaling Technology, 4060), anti-Akt (Cell Signaling Technology, 4691), anti-p-S9-Gsk3β (Cell Signaling Technology, 5558), anti-Gsk3 (Cell Signaling Technology, 5676), anti-p-S380/T382/T383-Pten (Cell Signaling Technology, 9554), anti-β-catenin (Cell Signaling Technology, 8480), anti-Stat3 (Santa Cruz, sc483), anti-Rex1 antibody (Abcam, ab175429), anti-Gata4 antibody (Abcam, ab84593), anti-Mef2c antibody (Cell Signaling Technology, 5030), anti-p-Y705-Stat3 (Cell Signaling Technology, 9145), anti-β-Tubulin (Cell Signaling Technology, 2146), anti-p-T389-p70 S6 Kinase (Cell Signaling Technology, 9234), anti-p70 S6 Kinase (Cell Signaling Technology, 9202), anti-p-S235/236-S6 Ribosomal protein (Cell Signaling Technology, 4858), anti-S6 Ribosomal protein (Cell Signaling Technology, 2217), anti-c-Myc (Cell Signaling Technology, 5605), anti-c-Jun (Cell Signaling Technology, 9165), anti-Sox2 (Cell Signaling Technology, 14962), anti-p-S250/251-Sox2 (Cell Signaling Technology, 92186), and anti-Lamin A/C (Cell Signaling Technology, 4777).

### Flow cytometry

A total of 1 × 10^6^ cells (WT ESCs, passage number 29) were required for optimal performance. The cells were fixed by adding 100 µl of 4% PFA to each sample for 15 min and then permeabilized by adding 100 µl of PBS and 0.3% Triton-X 100. The cells were stained with primary and secondary antibodies. The primary antibody was anti-Rex1 antibody (Abcam, ab175429) and the secondary antibodies were donkey anti-mouse IgG (H + L) Secondary Antibody (Life Technologies, A16018) and donkey anti-mouse IgG H + L (TRITC) preadsorbed (Abcam, ab7058).

### Cell proliferation assay

The number of viable cells was determined by a 3-(4,5-dimethylthiazol-2-yl)-5-(3-carboxymethoxyphenyl)-2-(4-sulfophenyl)-2H-tetrazolium (MTS) assay with a CellTiter 96 aqueous Cell Proliferation Assay kit (Promega).

### Cell apoptosis assay

Cell apoptosis was detected by using a kit to the product manual (Invitrogen, V13241).

### Real-time PCR analysis

Total RNA was isolated using Trizol (Invitrogen), and 500 ng of RNA was reverse-transcribed using a High-Capacity cDNA Reverse Transcription Kit (Applied Biosystems). mRNA expression was measured by SYBR Green quantitative PCR using the ^ΔΔ^Ct method. Gapdh was used for normalization. Technical replicates were carried out for all quantitative PCR reactions, and the primer sequences are listed in Table S1.

### RNA-seq

RNA-seq was performed for WT, *Pten*^*−/−*^, and *Pten*-3A mutant ESCs and for WT and *Pten*^*−/−*^ EBs. Total RNA was isolated from mouse ESCs and EBs according to the manufacturer’s instructions using TRIzol reagent. The RNA was converted into a template molecule library for sequencing on the BGISEQ-500 (BGI, ShenZhen, China). Kyoto encyclopedia of genes and genomes pathway enrichment analysis was performed by the DAVID Functional Annotation Clustering Tool (DAVID 6.8, https://david.ncifcrf.gov).

### Statistics

All statistical analyses were conducted using GraphPad Prism (version 6). Statistical significance was calculated by Student’s *t*-test between the indicated groups. Significance was defined by *p < 0.05; **p < 0.01; and ***p < 0.001. All experiments were performed independently at least three times.

## Supplementary information

Supplementary Figure Legends

Supplementary Figure 1

Supplementary Figure 2

Supplementary Figure 3

Supplementary Figure 4

Supplementary Figure 5

Supplementary Figure 6

Supplementary Figure 7

Supplementary Figure 8

Supplementary Figure 9

Supplementary Methods

Supplementary Tables

## Data Availability

The RNA-seq raw data and normalized mapped reads are available from the Gene Expression Omnibus (GEO) at accession number: GSE117280. All other data supporting the findings of this study are available from the corresponding author on reasonable request.
