# Supplementary material for: Pten-mediated Gsk3β modulates the naïve pluripotency maintenance in embryonic stem cells
Source: Cell Death Dis. 2020 Feb 7;11(2):107. doi: 10.1038/s41419-020-2271-0 (PMC7007436; doi:10.1038/s41419-020-2271-0)
Supplement: Supplementary file 1 — Supplementary Figure Legends [file 41419_2020_2271_MOESM1_ESM.docx]

**Supplementary Figure Legends**

**Fig. S1 *Pten* deletion maintained the naïve pluripotency of ESCs. Data related to Fig. 1**

(**a**) *Pten* deletion in mouse ESCs by CRISPR-Cas9. G1, G2, G3, G4, and G5 are different *Pten*^−/−^ cell lines, and G1 was utilized to perform experiments.

(**b**) qRT-PCR analysis of pluripotency markers (*Nanog*, *Oct4*, and *Klf4*) mRNA expression in wild-type (WT) and *Pten^−/−^* ESCs. Error bars indicate mean ± SD (n = 3).

(**c**) Phase-contrast images of WT and *Pten^−/−^* ESCs (G2, G3, and G4). *Pten^−/−^* ESCs had a greater proportion in the ground state. Scale bars, 100 µm.

(**d**) Western blot analysis showed that the expression of pluripotency markers (*Nanog*, *Oct4*, and *Klf4*) was increased in *Pten^−/−^* ESCs (G2, G3, and G4).

(**e**) Re-expression of Pten (Re-Pten) in *Pten^−/−^* ESCs restored the expression of Nanog, Klf4, and Oct4.

(**f**) Scatter plots of transcript expression in WT and *Pten^−/−^* ESCs. The expression values are shown on a log10 scale. Yellow dots indicate up-regulated genes in *Pten^−/−^* ESCs, and blue dots indicate down-regulated genes. Probability ≥ 0.8 and abs (log2(Y/X)) ≥ 1.

(**g**) PCA of WT and *Pten^−/−^* ESCs transcriptomes.

**Fig. S2 *Pten^−/−^* promoted the expression of naïve pluripotency genes. Data related to Fig. 1**

(**a**) Heat map of FPKM values of naïve and primed pluripotency genes in WT and *Pten^−/−^* ESCs. The heat map was normalized with sigma-normalization per row. See also Table S5.

(**b**) qRT-PCR analysis of primed pluripotency markers (*Otx2*, *Pou3f1*, *Lefty1*, *Lin28a*, and *Dnmt3b*) mRNA expression in WT and *Pten^−/−^* ESCs. Error bars indicate mean ± SD (n = 3).

(**c**) Western blot analysis of WT and *Pten^−/−^* ESCs showing the expression of Rex1.

(**d**) Proliferation ability of WT and *Pten^−/−^* ESCs. Error bars indicate mean ± SEM (n = 3).

(**e**) WT and *Pten^−/−^* ESCs were stained with Propidium iodide (red) and Hoechst (blue). Scale bars, 100 µm.

(**f**) Cell apoptosis assay for WT and *Pten^−/−^* ESCs. Q2 = necrotic cells, Q3 = viable cells, Q4 = apoptotic cells.

**Fig. S3 *Pten^−/−^* regulated ESC differentiation. Data related to Fig. 1**

(**a**) Western blot analysis of WT and *Pten^−/−^* EBs from day 0 to day 9 shows the expression of the pluripotency markers Nanog and Oct4.

(**b**) qRT-PCR analysis of differentiation markers (*Fgf5*, *Nestin*, *Prom1*, *Msi1*, *Brachyury*, *Bmp4*, *Gsc*, *Hind1*, *Gata4*, *Gata6*, *Sox17*, *Hnf4*, *and Afp*) mRNA expression in WT and *Pten^−/−^* ESCs with RA treatment. Error bars indicate mean ± SD (n = 3).

(**c**) Western blot analysis of WT and *Pten^−/−^* ESCs treated with RA showing the expression of the differentiation markers Mef2c and Gata4.

(**d**) Proliferation ability of WT and *Pten^−/−^* ESCs treated with RA. Error bars indicate mean ± SEM (n = 3).

(**e**) Cell apoptosis assay for WT and *Pten^−/−^* ESCs treated with RA. Q2 = necrotic cells, Q3 = viable cells, Q4 = apoptotic cells.

(**f**) WT and *Pten^−/−^* ESCs were injected into immunodeficient mice and produced teratocarcinomas containing tissues representative of the three germ layers (mesoderm, ectoderm, and endoderm).

(**g**) qRT-PCR analysis of early and mature markers at day 9 of ectoderm (*Pax6*, *Sox2*, *Tubb3*, and *Krt8*), mesoderm (*Mef2c*, *Kdr*, *MyoD1*, and *Mlc2v*), and endoderm (*Afp*, *Gata4*, and *FoxA2*). Error bars indicate mean ± SD (n = 3).

(**h**) Western blot analysis of WT and *Pten^−/−^* ESCs from day 0 to day 9 showing the expression of ectoderm marker (Pax6), mesoderm marker (Mef2c), and endoderm marker (Gata4).

(**i**) Phase-contrast images of EBs derived from WT and *Pten^−/−^* ESCs on day 4. Scale bars, 200 µm. Error bars indicate mean ± SD (n = 3). See also Table S6.

**Fig. S4** **The gap in ground pluripotency between WT and *Pten^−/−^* ESCs disappeared when cultured in medium with high concentrations of CHIR99021. Data related to Fig. 2**

(**a**) Phase-contrast images of WT and *Pten^−/−^* ESCs cultured in medium with different concentrations of CHIR99021 from 0 µM to 5 µM. Scale bars, 100 µm.

(**b**) AP staining analysis of WT and *Pten^−/−^* ESC colonies cultured in medium with different concentrations of CHIR99021. Error bars indicate mean ± SEM (n = 3), and 80 colonies were scored in each replicate.

(**c**) Western blot analysis of WT and *Pten^−/−^* ESCs cultured in medium without CHIR99021 showing the expression of the pluripotency markers *Nanog*, *Oct4*, and *Klf4*.

(**d**) Western blot analysis of WT and *Pten^−/−^* ESCs cultured in medium with 2iL showing the phosphorylation of Akt at T308 and S473 and the phosphorylation of Gsk3β at S9.

(**e**) Western blot analysis of WT and *Pten^−/−^* ESCs showing the total level and phosphorylation level of Sox2.

(**f**) Western blot analysis of WT and *Pten^−/−^* EBs at Day 7 showing the total level and phosphorylation level of Sox2.

(**g**) The phosphorylation levels of Gsk3β in WT and *Pten^−/−^* ESCs at different time points after removal of CHIR99021.

(**h**) Western blot analysis of WT and *Pten^−/−^* ESCs cultured in medium with different concentrations of CHIR99021 showing the expression of the pluripotency markers Nanog, Oct4, and Klf4.

(**i**) qRT-PCR analysis of the expression of Wnt-target genes (*c-Myc*, *Jun*, *Dkk1*, *Axin2*, *and Sp5*) in WT and *Pten^−/−^* ESCs. Error bars indicate mean ± SD (n = 3).

(**j**) Western blot analysis of the expression of Wnt-target genes in WT and *Pten^−/−^* ESCs.

**Fig. S5 Akt/mTOR pathway was involved in ESCs maintenance regulated by Pten. Data related to Fig. 2**

(**a**) Phase-contrast images of WT and *Pten^−/−^* ESC cultured in different concentrations of LIF. Scale bars, 100 µm.

(**b**) AP staining analysis of WT and *Pten^−/−^* ESC colonies cultured in medium with different concentrations of LIF from 0 units/ml to 5 × 10^3^ units/ml. Error bars indicate mean ± SEM (n = 3), and 80 colonies were scored in each replicate.

(**c**) Western blot analysis of WT and *Pten^−/−^* ESCs cultured in 2iL showing the phosphorylation of Akt at T308 and S473 and the phosphorylation of Stat3 at Y705.

(**d**) Western blot analysis of WT and *Pten^−/−^* ESCs showing the phosphorylation of p70S6K and S6.

(**e**) Western blot analysis of *Pten^−/−^* ESCs treated with mTOR-inhibitor rapamycin showing the restoration of the expression of Klf4 and Nanog.

**Fig. S6 The Pten-inhibitor SF1670 maintained ESC pluripotency. Data related to Fig. 3**

(**a**) Phase-contrast images of WT ESCs treated with different concentrations of the Pten-inhibitor SF1670 when cultured in mediums with 2iL. Scale bars, 100 µm.

(**b**) Phase-contrast images of WT ESCs, SF1670-treated ESCs, and *Pten^−/−^* ESCs when culture in medium with 2iL. Scale bars, 100 µm.

(**c**, **d**) Analysis of AP staining of WT ESC colonies, SF1670-treated ESC colonies, and *Pten^−/−^* ESC colonies. Error bars indicate mean ± SEM (n = 3), and 80 colonies were scored in each replicate.

(**e**) Immunofluorescence staining for Nanog in WT ESCs treated with DMSO and SF1670 cultured in medium with 2iL. Scale bars, 50 µm.

**Fig. S7 Pten-inhibitor SF1670 could replace the Gsk3β-inhibitor CHIR99021 in ESC maintenance. Data related to Fig. 4**

(**a**) Phase-contrast images of DMSO-treated and SF1670-treated WT ESCs cultured in medium including 2iL and in medium lacking PD0325901, LIF, CHIR99021, 2i, and 2iL, respectively. Scale bars, 100 µm.

(**b**) AP staining of DMSO-treated and SF1670-treated WT ESCs cultured in medium including 2iL and in medium lacking PD0325901, LIF, CHIR99021, 2i, and 2iL, respectively. Scale bars, 100 µm.

(**c**) Phase-contrast images of WT ESCs treated with different concentrations of the Pten-inhibitor SF1670 when cultured in media without CHIR99021. Scale bars, 100 µm.

(**d**) Immunofluorescence staining for Nanog in DMSO-treated and SF1670-treated WT ESCs cultured in medium without CHIR99021. DNA was stained with DAPI to indicate nuclei. Scale bars, 50 µm.

(**e**) Western blot analysis of WT ESCs treated with DMSO and SF1670 showing the expression of Nanog, Klf4, and Oct4 in the absence of LIF.

(**f**) Western blot analysis of WT ESCs treated with SF1670 showing the expression of Rex1.

(**g**) The proportion of Rex1 positive cells was analyzed for the DMSO-treated and SF1670-treated ESCs by flow cytometry.

(**h**) Proliferation ability of WT ESCs treated with DMSO and SF1670. Error bars indicate mean ± SEM (n = 3).

(**i**) Cell apoptosis assay for WT ESCs treated with DMSO and SF1670. Q2 = necrotic cells, Q3 = viable cells, Q4 = apoptotic cells.

**Fig. S8** **The Pten S380A, T382A, T383A (Pten-A3) mutations suppressed the pluripotency of ESCs. Data related to Fig. 5**

(**a**) Schematic of the sgRNA/targeting template-targeting site at *Pten* exon 9. The targeting template contained 937 bp and 1026 bp homology arms flanking the DSBs on both sides. Pten S380A, T382A, and T383A mutations were confirmed by sequencing.

(**b**) Western blots analysis showing the loss of phosphorylation in another two Pten-A3 mutants.

(**c**) Overexpression of Pten (O-Pten) in Pten-A3 mutant ESCs restored the expression of Klf4 and Oct4.

(**d**) Immunofluorescence staining for Nanog in WT, Pten-A3 mutant, and *Pten^−/−^* ESCs. DNA was stained with DAPI to indicate nuclei. Scale bars, 50 µm.

(**e**) Phase-contrast images of WT, Pten-A3 mutant, and *Pten^−/−^* ESCs. Scale bars, 100 µm.

(**f**) AP staining of WT, Pten-A3 mutant, and *Pten^−/−^* ESC colonies cultured for 4 days. Flattened colonies are indicated by black arrows, and domed colonies are indicated by green arrows. Scale bars, 100 µm.

(**g**) Analysis of colony morphology of WT, Pten-A3 mutant, and *Pten^−/−^* ESC colonies. Error bars indicate mean ± SEM (n = 3), and 80 colonies were scored in each replicate.

(**h**) Proliferation ability of WT and Pten-A3 mutant ESCs. Error bars indicate mean ± SEM (n = 3).

**Fig. S9 *Pten^−/−^* ESCs showed a bias towards ectoderm differentiation in EBs.** **Data related to Fig. 6**

(**a**) PCA of transcriptomes of different EBs derived from WT and *Pten^−/−^* ESCs.

(**b**) Pearson correlation coefficients for all gene expression of WT and *Pten^−/−^* EBs.

(**c**) qRT-PCR analysis of WT and *Pten^−/−^* EBs showing the expression of ectoderm markers.

(**d**) Signaling pathway analyzed by David functional annotation for the transcriptome of WT and *Pten^−/−^* EBs.

(**e**) Signaling pathway analyzed by David functional annotation for the transcriptome of WT and *Pten^−/−^* ESCs.

(**f**) Signaling pathway analyzed by David functional annotation for the transcriptome of WT and Pten-A3 ESCs.
