# Supplementary figures and images for: Pten-mediated Gsk3β modulates the naïve pluripotency maintenance in embryonic stem cells
Source: Cell Death Dis. 2020 Feb 7;11(2):107. doi: 10.1038/s41419-020-2271-0 (PMC7007436; doi:10.1038/s41419-020-2271-0)

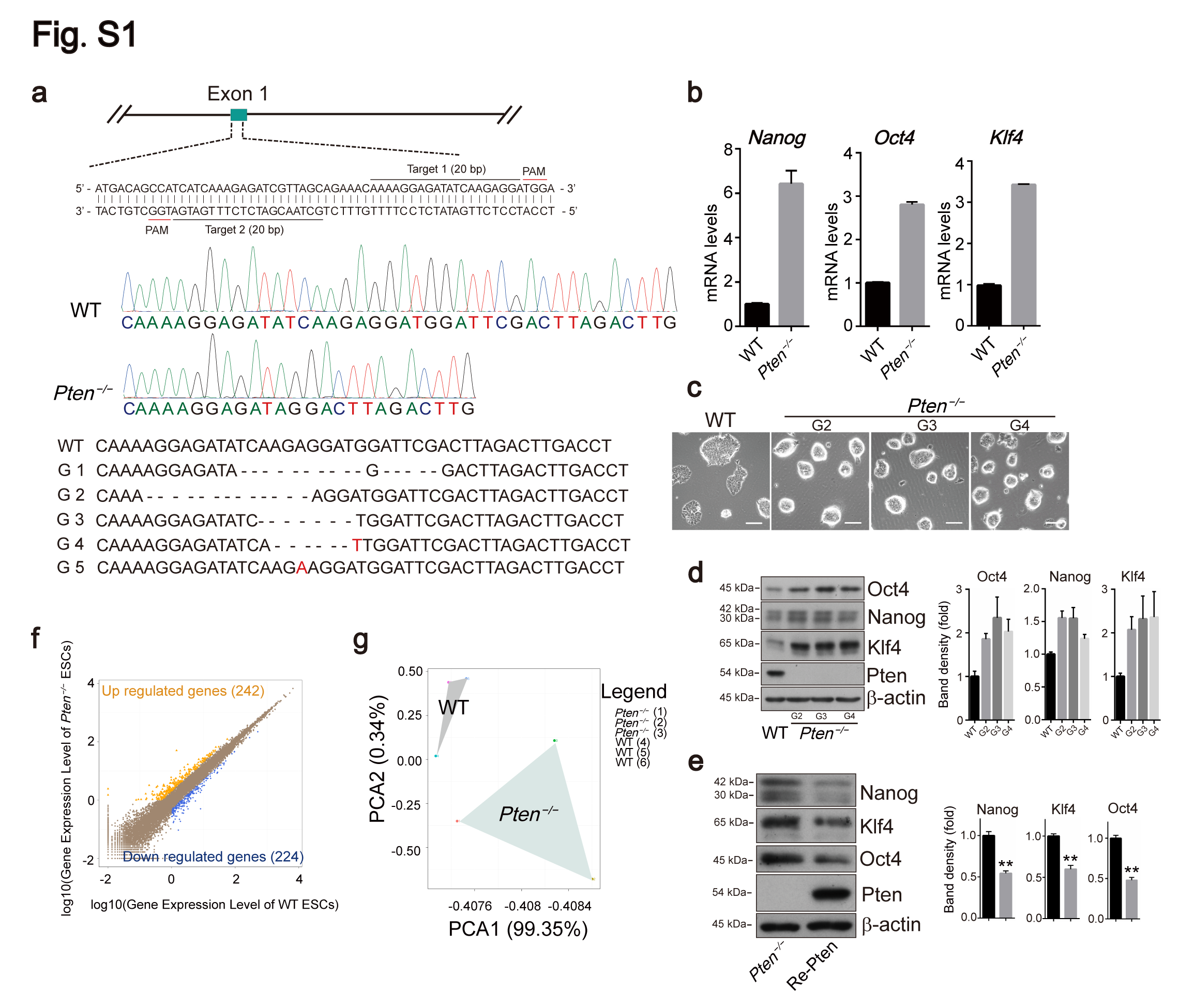

Supplement: Supplementary file 2 — Supplementary Figure 1 [file 41419_2020_2271_MOESM2_ESM.tif]

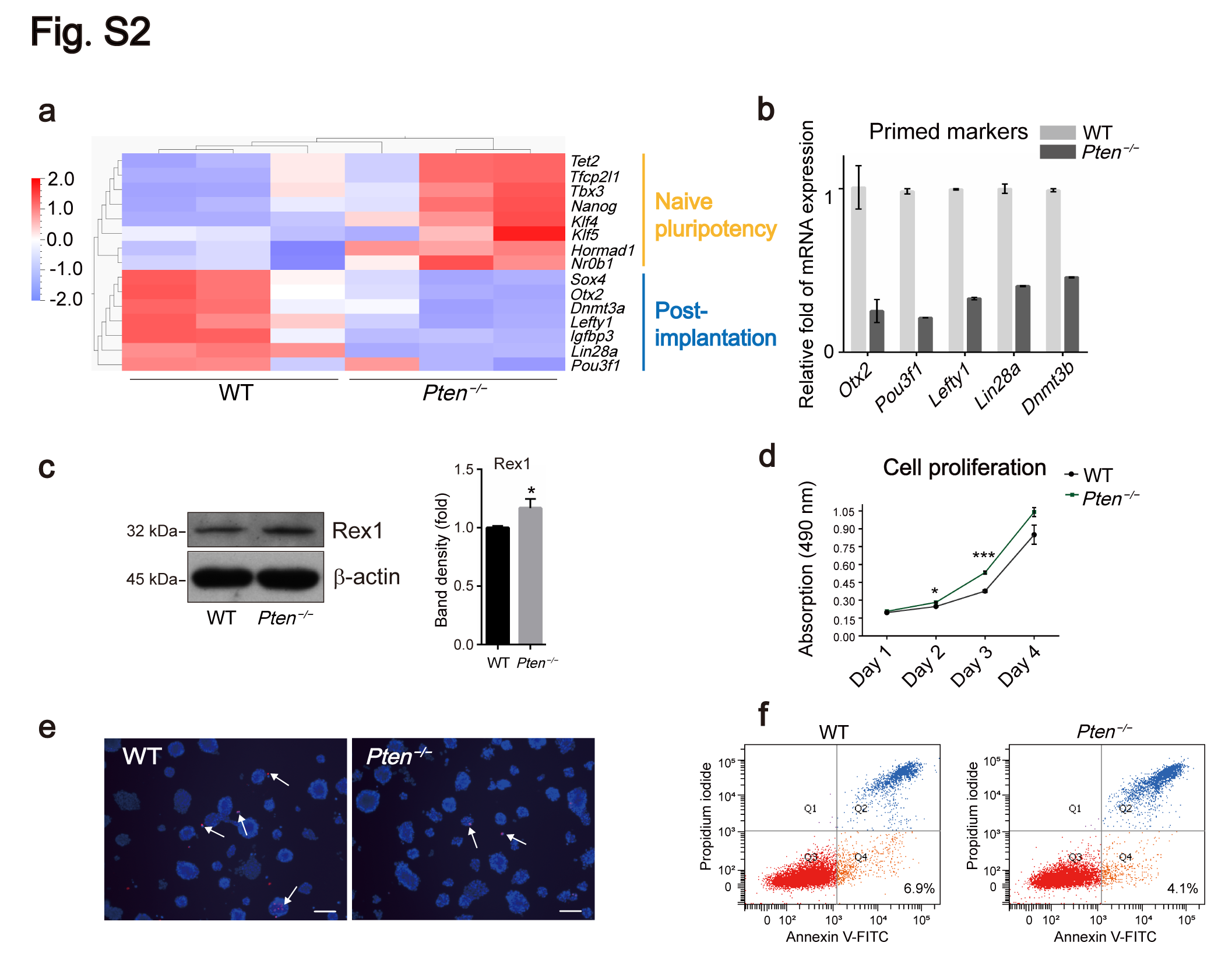

Supplement: Supplementary file 3 — Supplementary Figure 2 [file 41419_2020_2271_MOESM3_ESM.tif]

Fig. S3

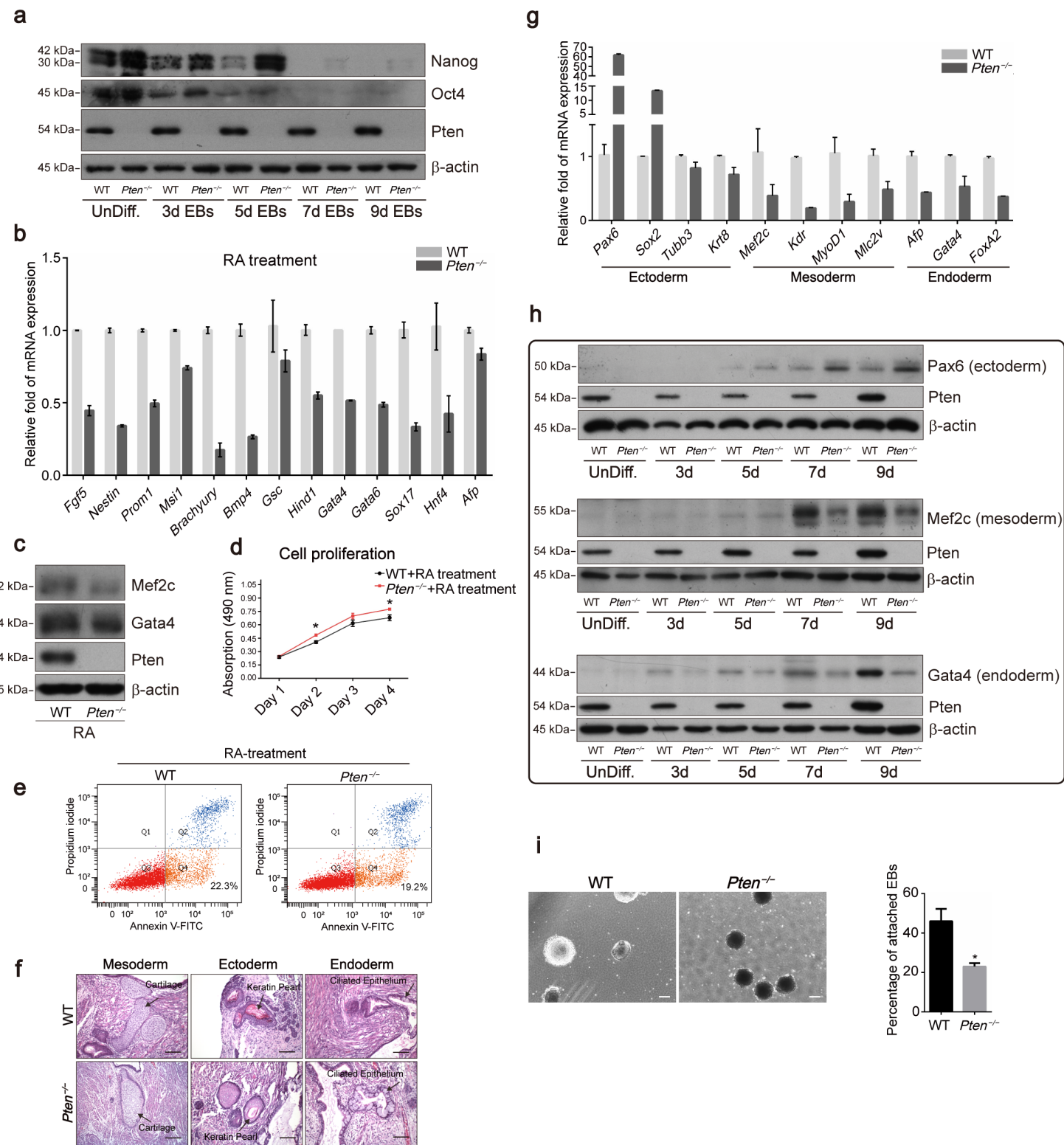

Supplement: Supplementary file 4 — Supplementary Figure 3 [file 41419_2020_2271_MOESM4_ESM.pdf]

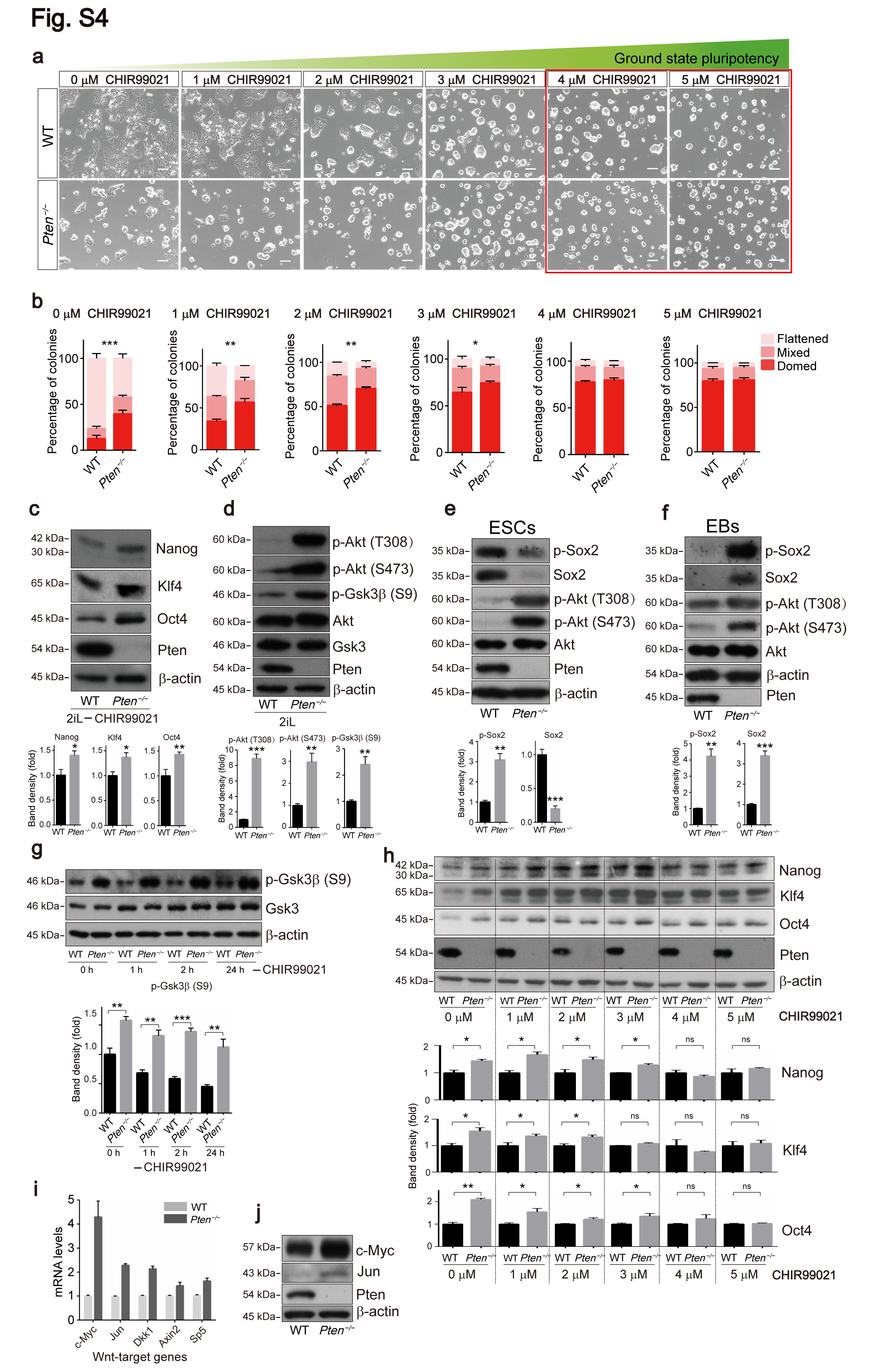

Supplement: Supplementary file 5 — Supplementary Figure 4 [file 41419_2020_2271_MOESM5_ESM.tif]

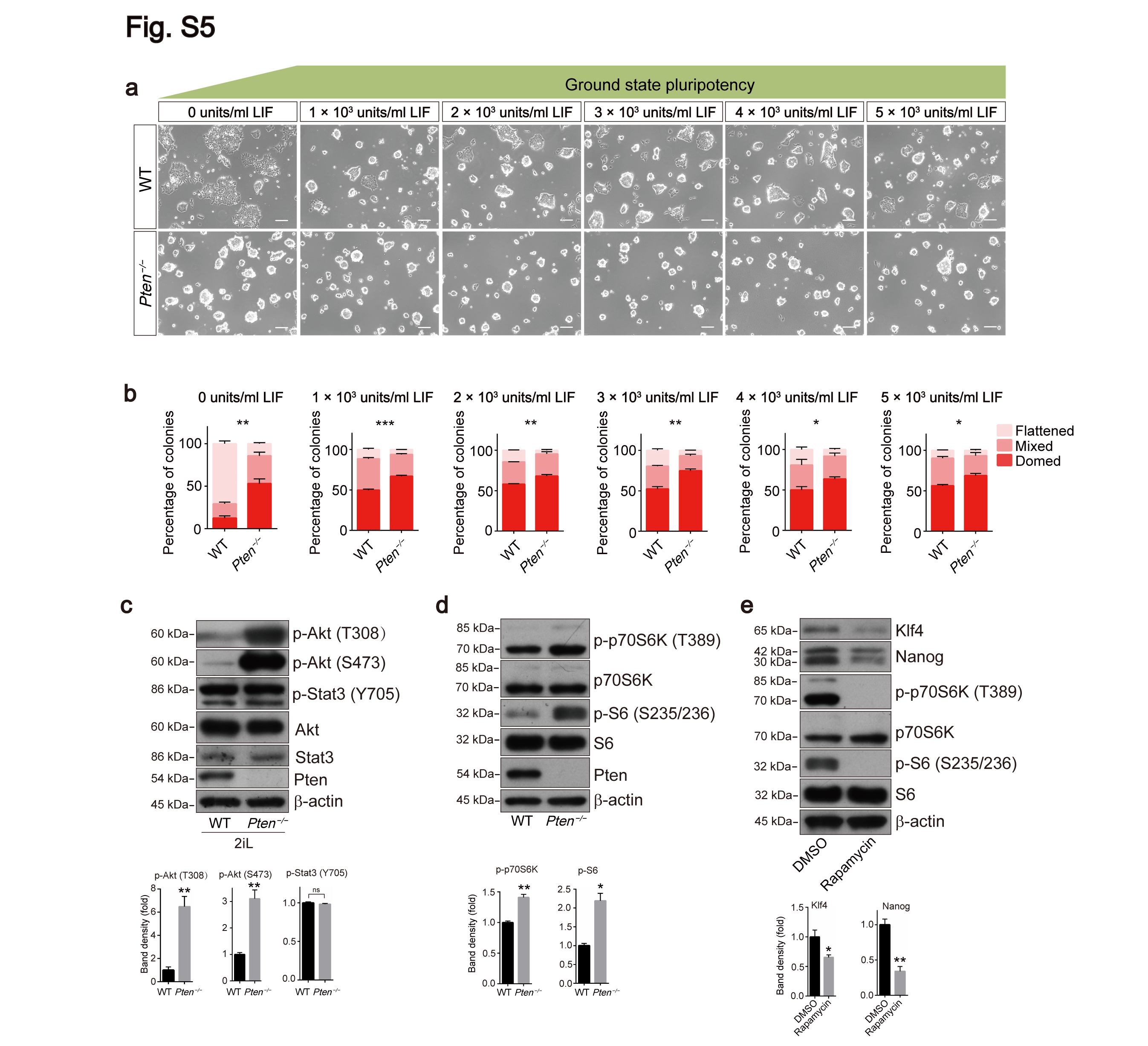

Supplement: Supplementary file 6 — Supplementary Figure 5 [file 41419_2020_2271_MOESM6_ESM.tif]

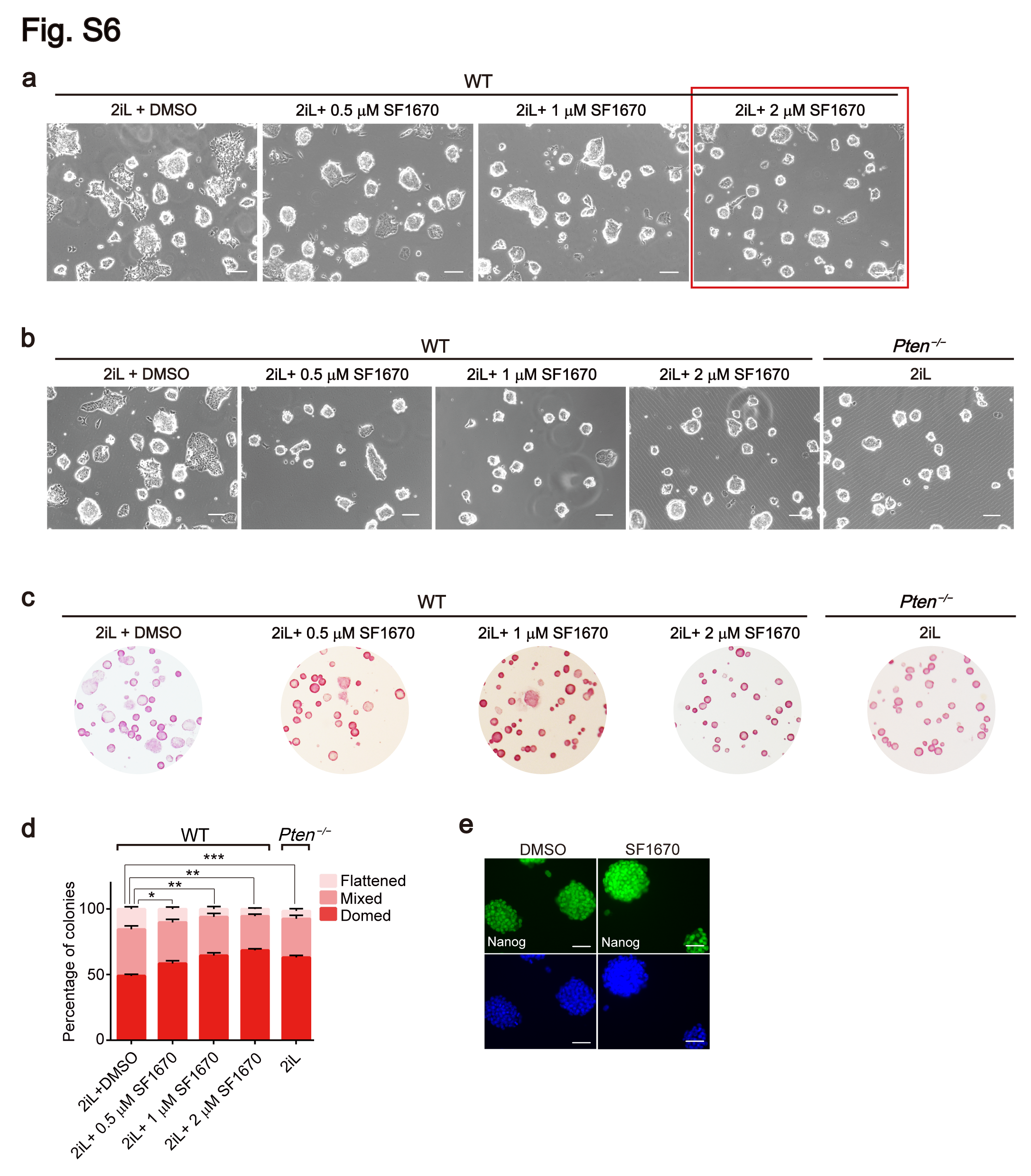

Supplement: Supplementary file 7 — Supplementary Figure 6 [file 41419_2020_2271_MOESM7_ESM.tif]

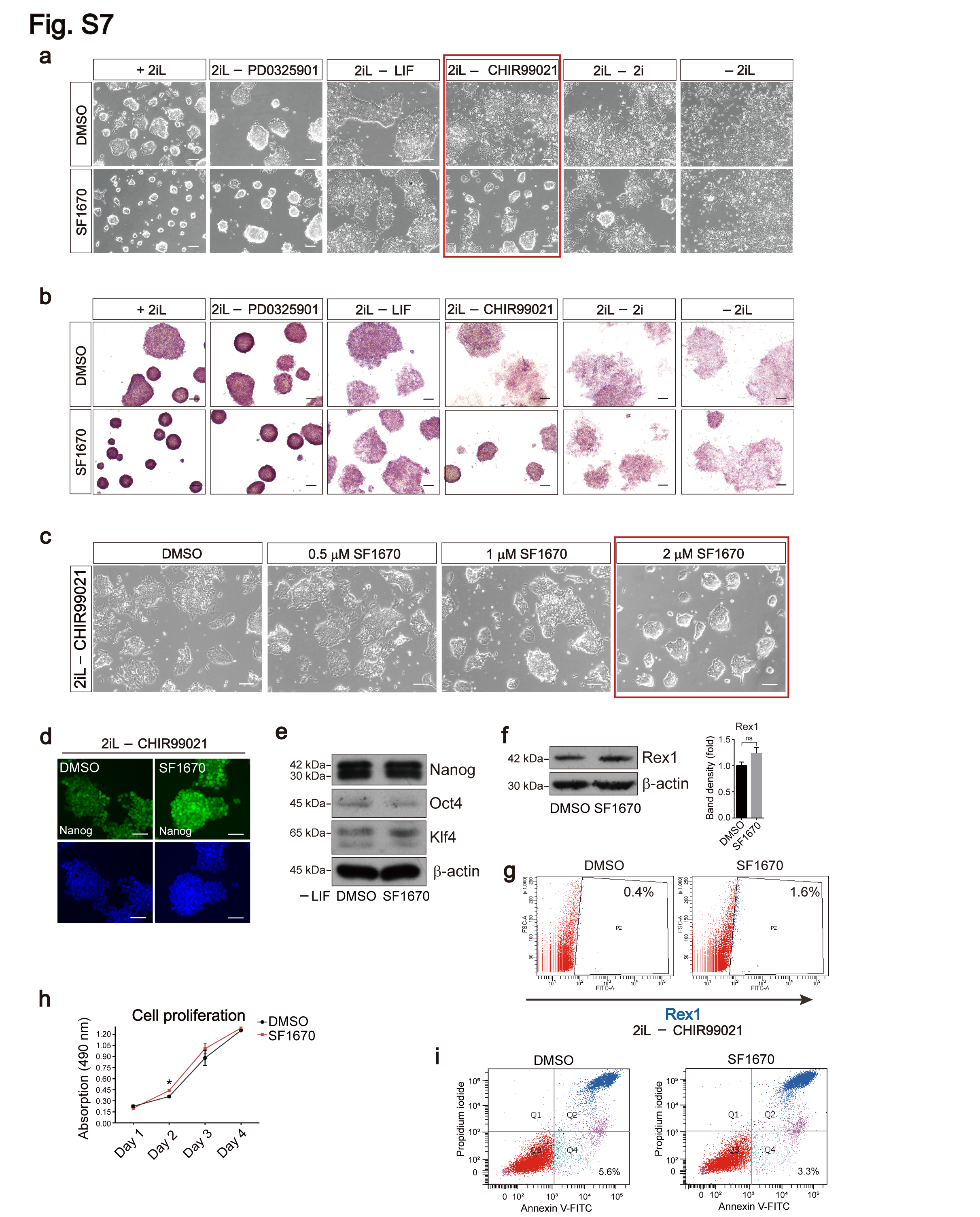

Supplement: Supplementary file 8 — Supplementary Figure 7 [file 41419_2020_2271_MOESM8_ESM.tif]

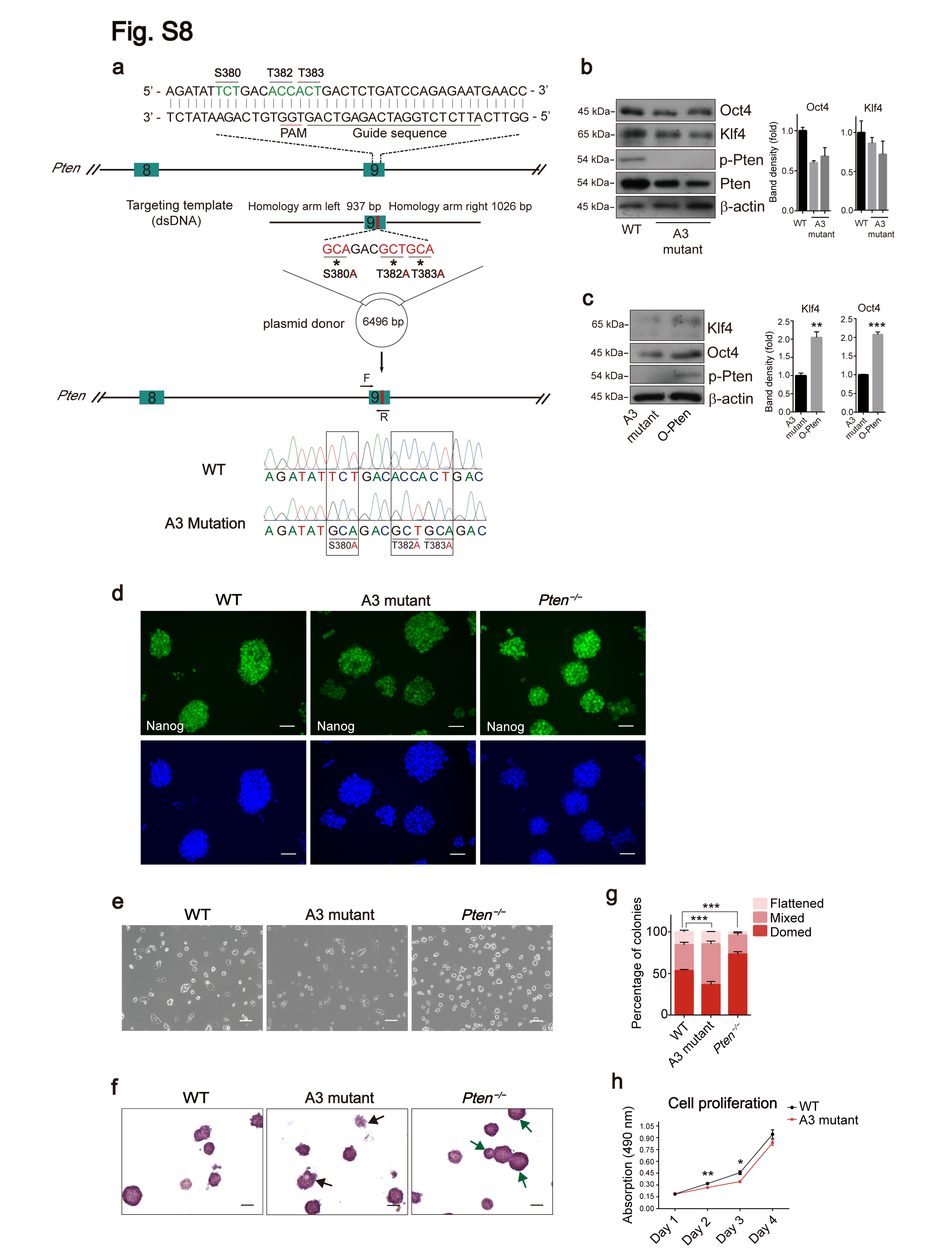

Supplement: Supplementary file 9 — Supplementary Figure 8 [file 41419_2020_2271_MOESM9_ESM.tif]

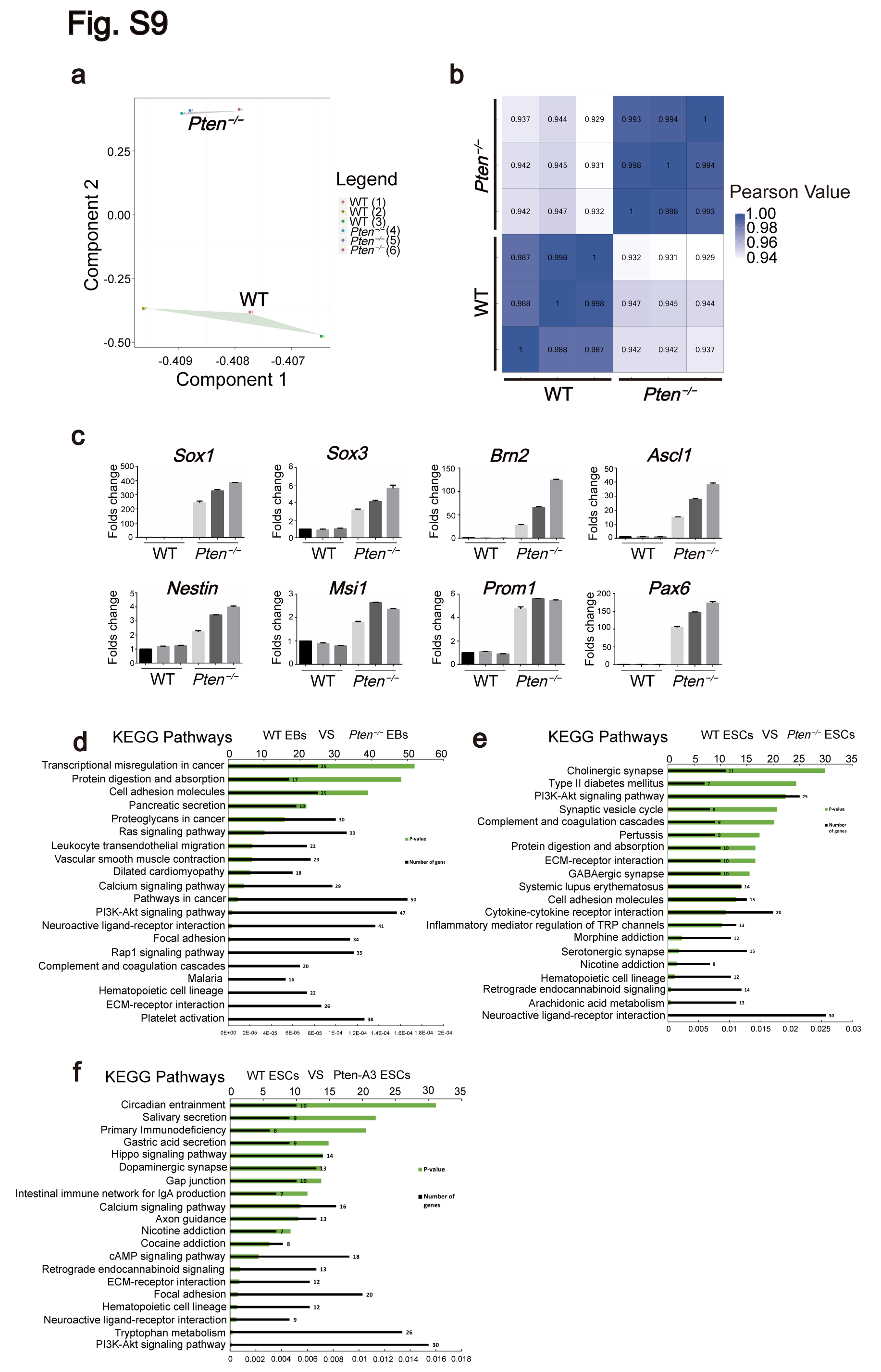

Supplement: Supplementary file 10 — Supplementary Figure 9 [file 41419_2020_2271_MOESM10_ESM.tif]
