# Supplementary material for: Pten-mediated Gsk3β modulates the naïve pluripotency maintenance in embryonic stem cells
Source: Cell Death Dis. 2020 Feb 7;11(2):107. doi: 10.1038/s41419-020-2271-0 (PMC7007436; doi:10.1038/s41419-020-2271-0)
Supplement: Supplementary file 11 — Supplementary Methods [file 41419_2020_2271_MOESM11_ESM.docx]

**Supplementary Methods**

***Pten* deletion in mouse ESCs by CRISPR-Cas9 technologies**

Guide sequences of sgRNA were incorporated into the pSpCas9(BB)-2A-GFP vector (Addgene, 48138) containing *Cas9* and green fluorescent protein (*GFP*) genes. ESCs (passage number 24) were transfected with constructs containing *Cas9* and target sequences using lipofectamine 3000, and these were then subjected to cell sorting of GFP-positive cells after transfection for 48 h. The enriched gene-modified cell populations were cultured in 60-mm dishes for 4-5 days, and each cell clone was passaged into 24-well plates. Finally, the *Pten^−/−^* cell lines were identified by sequencing, and the cells were stored in liquid nitrogen.

**Pten-A3 mutations in mouse ESCs by CRISPR-Cas9 technologies**

The sgRNA sequence for site-directed mutagenesis was designed as shown in Table S1 and was incorporated into the BbsI site of the pSpCas9 (BB)-2A-GFP vector. The targeting template for the Pten S380A, T382A, and T383A mutations in the mouse *Pten* locus was made by overlap PCR with primers *Pten* S380A-T382A-T383A donor For-1, *Pten* S380A-T382A-T383A donor Rev-1, *Pten* S380A-T382A-T383A donor For-2, and *Pten* S380A-T382A-T383A donor Rev-2. The PAM was modified on the template to avoid DSBs on the plasmid donor. The donor template contained a 937 bp homology arm and a 1026 bp homology arm flanking the DSBs on both sides and was incorporated into the EcoRV/XbaI sites of the CD44P pGL3 vector (Addgene, 19122). Single clone cells were cultured in a 24-well plate and stored in liquid nitrogen (Fig. S8a). The presence of a mutated cassette in the *Pten* locus was analyzed using an external forward primer (*Pten* S380A-T382A-T383A screen For-1) specific for a sequence outside of the flanking arm and an internal reverse primer (*Pten* S380A-T382A-T383A screen Rev-2) specific to the mutated cassette (Fig. S8a). The potential mutant DNA fragments were amplified for sequencing with primers *Pten* S380A-T382A-T383A screen For-1 and *Pten* S380A-T382A-T383A screen Rev-1. The sequences of the primers are shown in Table S1.

**Construction of vector**

CDS sequence of Pten was amplified by PCR using Platinum pfx DNA Polymerase and inserted into the XbaI and BamHI sites of a lentivirus vector, PCDH vector (System Biosciences, # CD513B-1). The primer information was described in Table S1.

**Three germ layer differentiation**

Ectoderm differentiation protocol was modified from a previous report [^1^](file:///D:\PTEN%20knock%20out\data\Ai%20files\For%20submit%2020180321\submit%20format%2020180425\small%20CDD%2020190609\Revise%20files\Revised%20manuscript%20and%20comments\Supplemental%20Information.docx#_ENREF_1). In brief, EBs were formed using the hanging drop method with 250 cells/drop in DMEM/F12 medium containing GlutaMAX and sodium pyruvate with 15% fetal bovine serum, nonessential amino acid solution, and β-[mercaptoethanol](http://dict.youdao.com/w/mercaptoethanol/#keyfrom=E2Ctranslation). After 3 days of hanging drop culture, EBs were collected and transferred to petri dishes in medium with Retinoic acid (RA). After 2 days, the EBs were transferred to tissue culture plate for attachment culture in medium with β-Glycerol phosphate.

Endoderm differentiation protocol was modified from a previous report [^2^](file:///D:\PTEN%20knock%20out\data\Ai%20files\For%20submit%2020180321\submit%20format%2020180425\small%20CDD%2020190609\Revise%20files\Revised%20manuscript%20and%20comments\Supplemental%20Information.docx#_ENREF_2). In brief, EBs were formed as the above description, and EBs were transferred to Petri dish in endoderm induction medium containing DMEM/F12 media, GlutaMAX, sodium pyruvate, 15% fetal bovine serum, nonessential amino acid solution, β-[mercaptoethanol](http://dict.youdao.com/w/mercaptoethanol/#keyfrom=E2Ctranslation), 0.6% BSA, 20 ng/ml FGF2, 20 ng/ml Activin A, after 2 days, the cells were transferred to tissue culture plate for attachment culture in endoderm induction medium.

For mesoderm differentiation, cells were suspended in modified serum-free differentiation medium containing DMEM/F12, B27, N2 supplements, GlutaMAX, 10% BSA, and L-ascorbic acid at a final concentration of 75,000 cells/mL in non-adherent Petri dish, without any additional [growth factors](https://www.sciencedirect.com/topics/biochemistry-genetics-and-molecular-biology/growth-factors) for 2 days. Then EBs were dissociated, and the cells were reaggregated in the presence of recombinant [Activin](https://www.sciencedirect.com/topics/biochemistry-genetics-and-molecular-biology/activin) A, Dkk1, and BMP4 for 2 days. The fresh medium was replaced every other day.

**ESCs treated with AKT inhibitors**

The *Pten^−/−^* ESCs were cultured in CHIR99021-deficient medium and treated with 20 µM of LY294002 (Cell Signaling Technology, 9901) and 2 µM of PX-866 (BioVision, 1965).

**ESCs treated with SF1670**

The WT ESCs (passage number 28) were cultured in ESC culture medium with and without CHIR99021. After plating for 12 h, the cells were treated with 0.5 µM, 1 µM, and 2 µM concentration of SF1670 (Sigma, SML0684), respectively. The fresh medium was replaced every other day.

**Library preparation for RNA-seq**

Briefly, total RNA samples were ribo-depleted and then fragmented. The fragmented samples were reversed transcribed to cDNA, end-repaired and 3’ adenylated. Adaptors were ligated to the ends of these 3’ adenylated cDNA fragments. The ligation products were purified, and several rounds of PCR amplification were performed to enrich the purified cDNA template using PCR primers. The PCR products were heat denatured, and the single strand DNA was cyclized using splint oligo and DNA ligase. Finally, the samples were sequenced on the BGISEQ-500 platform.

**RNA-seq data processing**

The raw reads were cleaned using internal software SOAPnuke (Version v1.5.2, Website: https://github.com/BGI-flexlab/SOAPnuke). The clean reads were stored in the FASTAQ format. We used HISAT (Version v2.0.4, Website: <http://www.ccb.jhu.edu/software/hisat>) to do the mapping step, and we calculated gene expression with a software package called RSEM (Version v1.2.12, Website: http://deweylab.biostat.wisc.edu/ RSEM). The FPKM method was used to calculate the expression level, and the calculated gene expression can be directly used for comparing the difference in gene expression among the samples.

**Supplementary References**

1 Okada, Y., Shimazaki, T., Sobue, G. & Okano, H. Retinoic-acid-concentration-dependent acquisition of neural cell identity during in vitro differentiation of mouse embryonic stem cells. *Dev. Bio* **275**, 124-142, doi:10.1016/j.ydbio.2004.07.038 (2004).

2 Soto-Gutierrez, A. *et al.* Differentiation of mouse embryonic stem cells to hepatocyte-like cells by co-culture with human liver nonparenchymal cell lines. *Nat. Protocols* **2**, 347-356, doi:10.1038/nprot.2007.18 (2007).
