# Supplementary material for: Pten-mediated Gsk3β modulates the naïve pluripotency maintenance in embryonic stem cells
Source: Cell Death Dis. 2020 Feb 7;11(2):107. doi: 10.1038/s41419-020-2271-0 (PMC7007436; doi:10.1038/s41419-020-2271-0)
Supplement: Supplementary file 12 — Supplementary Tables [file 41419_2020_2271_MOESM12_ESM.docx]

**Table S1.** Lists of sgRNAs and primers.

| m*Dkk1* For | GATCTGTCTGGCTTGCCGAA |
| --- | --- |
| m*Dkk1* Rev | TCTGGGATATCCATCCCCCG |
| m*Axin2* For | GACAGCGAGTTATCCAGCGA |
| m*Axin2* Rev | CGGTGGGTTCTCGGAAAATG |
| m*Sp5* For | CGTTCGTCCTCGCCTTCGG |
| m*Sp5* Rev | ACCTGAAGGAAGTCGGGTGC |
| m*Krt8* For | AAGTTCGTGCCCAGTACGAG |
| m*Krt8* Rev | TTAATGGCCATCTCCCCACG |
| m*Tubb3* For | CTGGTGGACTTGGAACCTGG |
| m*Tubb3* Rev | CCGCACGACATCTAGGACTG |
| m*Kdr* For | ATAACCTGGCTGACCCGATT |
| m*Kdr* Rev | GGGGGATGGAGAAAATCGCC |
| m*Mef2c* For | CACGCATCTCACCGCTTGAC |
| m*Mef2c* Rev | ATTGGCCTTCCACGCTTCAC |
| m*Myod1* For | CCATCCGCTACATCGAAGGT |
| m*Myod1* Rev | GTAGTAGGCGGTGTCGTAGC |
| m*Dnmt3b* For | AGAATTTCAGGAGCCACCCAA |
| m*Dnmt3b* Rev | ATGGGCTTCCACCAATCACC |
| m*Otx2* For | TGGTTGGAGAGTTTGCGTCA |
| m*Otx2* Rev | TACAGCCGCATTGGACGTTA |
| m*Pou3f1* For | CTCACCTTTTCTCCGGGCTT |
| m*Pou3f1* Rev | ATACACAGATGCGGCTCTCG |
| m*Lefty1* For | GCTCGATCAACCGCCAGTC |
| m*Lefty1* Rev | GGTGAGTGGAGGTCTCTGAC |
| m*Lin28a* For | TTTGCCTCCGGACTTCTCTG |
| m*Lin28a* Rev | GCGCACGTTGAACCACTTAC |
| m*Sox1* For | TTACTTCCCGCCAGCTCTTC |
| m*Sox1* Rev | TGATGCATTTTGGGGGTATCTCTC |
| m*Nestin* For | CTGCAGGCCACTGAAAAGTT |
| m*Nestin* Rev | GACCCTGCTTCTCCTGCTC |
| m*Pax6* For | CTAAGGATGTTGAACGGGCA |
| m*Pax6* Rev | AGTTGGTGTTCTCTCCCCCT |
| m*Gata6* For | TGCAAGATTGCATCATGACAGA |
| m*Gata6* Rev | TGACCTCAGATCAGCCACGTTA |
| m*Sox17* For | TTCTGTACACTTTAATGAGGCTGTTC |
| m*Sox17* Rev | TTGTGGGAAGTGGGATCAAG |
| m*Gata4* For | CTGGAAGACACCCCAATCTC |
| m*Gata*4 Rev | CAGGCATTGCACAGGTAGTG |
| m*Brachyury* For | CATCGGAACAGCTCTCCAACCTAT |
| m*Brachyury* Rev | GTGGGCTGGCGTTATGACTCA |
| m*Gsc* For | TCCAGGAGACGAAGTACCCA |
| m*Gsc* Rev | TCGGCGTTTTCTGACTCCTC |
| m*Bmp4* For | CCTCTTCAACCTCAGCAGCATCC |
| m*Bmp4* Rev | CACACCCCTCTACCACCATCTCC |
| m*Fgf5* For | AAAGTCAATGGCTCCCACGAA |
| m*Fgf5* Rev | GGCACTTGCATGGAGTTTTCC |
| m*FoxA2* For | GAGCAGCAACATCACCACAG |
| m*FoxA2* Rev | CGTAGGCCTTGAGGTCCAT |
| m*Hand1* For | AAGGATGCACAAGCAGGTGAC |
| m*Hand1* Rev | TTTAATCCTCTTCTCGCCGGG |
| m*Afp* For | GAAACCTCCAGGCAACAACC |
| m*Afp* Rev | GTTTGACGCCATTCTCTGCG |
| m*Hnf4* For | TTTGATCCAGATGCCAAGGG |
| m*Hnf4* Rev | TTGCTTGGTGATCGTTGGCT |
| m*Flk1* For | AAGGAACTAGAATGCGGGCT |
| m*Flk1* Rev | ACTCCCTGCTTTTACTGGGC |
| m*Ascl1* For | TCCTACGACCCTCTTAGCCC |
| m*Ascl1* Rev | CATTTGACGTCGTTGGCGAG |
| m*Sox3* For | GTTTAGCTTTGCTCGCCTGG |
| m*Sox3* Rev | GGTTTCTCCGCAAACACCAC |
| m*Msi1* For | AAGAGTGTCTGGTGATGCGG |
| m*Msi1* Rev | TCCACCTTTCCGAACTGCTC |
| m*Brn2* For | CCACAGACATGCTGATGGGT |
| m*Brn2* Rev | AGCACGGAAGTTACCAGAGC |
| m*Prom1* For | AGTGGAAGGAGCCCAGCTTAG |
| m*Prom1* Rev | TCCACACAGCCCCAGTAACA |
| m*Nanog* For | AGGGTCTGCTACTGAGATGCTCTG |
| m*Nanog* Rev | CAACCACTGGTTTTTCTGCCACCG |
| m*Oct4* For | TGGACCTCAGGTTGGACTGG |
| m*Oct4* Rev | CCTTTCCAAAGAGAACGCCC |
| m*Klf4* Forw | CATTAATGAGGCAGCCACCTG |
| m*Klf4* Rev | GTGAGAGAGTTCCTCACGCC |
| m*Rexo1* For | TCCCAAAAGAATTTGGCGGC |
| m*Rexo1* Rev | CACTGGCTTTGCTGAGGTTG |
| m*Esrrb* For | GGATGCTGAAGGAAGGTGTGC |
| m*Esrrb* Rev | TGGGAGGCATAGCATACAGC |
| m*Lefty2* For | CCTGGACAGCGCGGATG |
| m*Lefty2* Rev | GGACAGCCTCTCAAACCTCC |
| m*Zfp42* For | CTGGGTACGAGTGGCAGTTT |
| m*Zfp42* Rev | GTGAGGCGATCCTGCTTTCT |
| m*Fgf4* For | GGTGAGCATCTTCGGAGTGG |
| m*Fgf4* Rev | ACCTTCATGGTAGGCGACAC |
| m*Pten* KO sgRNA For-1 | CACCGAAAAGGAGATATCAAGAGGA |
| m*Pten* KO sgRNA Rev-1 | AAACTCCTCTTGATATCTCCTTTTC |
| m*Pten* KO sgRNA For-2 | CACCGGCTAACGATCTCTTTGATGA |
| m*Pten* KO sgRNA Rev-2 | AAACTCATCAAAGAGATCGTTAGCC |
| m*Pten* KO screen For | CGCCAAGTCCAGAGCCATTTCCATC |
| m*Pten* KO screen Rev | CACTGGAAGAGACACAATATGCACG |
| m*Pten* S380A-T382A-T383A sgRNA For | CACCGATTCTCTGGATCAGAGTCAG |
| m*Pten* S380G-T382A-T383A sgRNA Rev | AAACCTGACTCTGATCCAGAGAATC |
| m*Pten* S380A-T382A-T383A donor For-1 | CCGGATATCGAACCCAGTCTTGATGTAGCAGTGT |
| m*Pten* S380A-T382A-T383A donor Rev-1 | GGATCAGAGTCTGCAGCGTCTGCATATCTATAATGATCAGGTTCATTG |
| m*Pten* S380A-T382A-T383A donor For-2 | GCAGACGCTGCAGACTCTGATCC AGAGAATGAACCTTTTGATGAAGAT |
| m*Pten* S380A-T382A-T383A donor Rev-2 | TGCTCTAGA AGCATCTGACTCTTAGCACTGGCCT |
| m*Pten* S380A-T382A-T383A screen For | AGTAGAGGAGCCATCAAATCCAGAG |
| m*Pten* S380A-T382A-T383A screen Rev | CAATCTGACACAATGTCCTATTGCC |
| m*Pten* S380A-T382A-T383A screen Reverse-2 | CTGGATCAGAGTCTGCAGCGTCTGC |
| m*Pten* S380A-T382A-T383A sgRNA For | CACCGATTCTCTGGATCAGAGTCAG |
| m*Pten* S380G-T382A-T383A sgRNA Rev | AAACCTGACTCTGATCCAGAGAATC |
| m*Pten*-PCDH For | TGCTCTAGAGCCACCATGACAGCCATCATCAAAGAGATCG |
| m*Pten*-PCDH Rev | CGCGGATCCTCAGACTTTTGTAATTTGTGAATGC |

|  | **WT** | **WT** | **WT** | ***Pten^−/−^*** | ***Pten^−/−^*** | ***Pten^−/−^*** |
| --- | --- | --- | --- | --- | --- | --- |
| **Gene Symbol** |  |  |  |  |  |  |
| *Nanog* | 284.71 | 231.25 | 213.47 | 430.91 | 409.68 | 273.49 |
| *Oct4* | 588.7 | 559.68 | 564.99 | 616.3 | 658.21 | 659.01 |
| *Klf4* | 58.05 | 53.79 | 54.03 | 88.08 | 80.03 | 72.49 |
| *Nr0b1* | 28.02 | 37.38 | 36.79 | 50.15 | 54.99 | 42.78 |
| *Fgf4* | 38.82 | 36.8 | 38.85 | 41.84 | 42.71 | 55.12 |
| *Gm7325* | 34.74 | 33.42 | 31.65 | 43.12 | 45.41 | 51.11 |
| *Mta2* | 67.81 | 59.07 | 62.49 | 76.56 | 80.48 | 71.91 |
| *Parp1* | 183.37 | 161.53 | 163.44 | 191 | 192.51 | 170.96 |
| *Kdm5b* | 46.78 | 47.66 | 47.72 | 53.61 | 55.13 | 49.96 |
| *Sox15* | 5.6 | 8.49 | 5.5 | 10.11 | 9.57 | 12.2 |
| *Zfp57* | 254.2 | 251.63 | 244.74 | 291.4 | 297.34 | 281.91 |
| *E2f1* | 15.61 | 14.93 | 16.55 | 26.02 | 22.7 | 25.89 |
| *Gmnn* | 68.33 | 68.02 | 65.97 | 75.9 | 86.94 | 100.17 |
| *Tfcp2l1* | 66.45 | 32.24 | 32.4 | 96.65 | 96.24 | 44.16 |
| *Cd9* | 156.69 | 121.07 | 130.1 | 165.45 | 157.29 | 166.93 |
| *Nodal* | 16.51 | 14.2 | 16.71 | 19.43 | 18.96 | 15.79 |
| *Ifitm2* | 293.55 | 293.23 | 283.05 | 335.71 | 309.75 | 324.86 |
| *Tet2* | 44.5 | 35.33 | 33.08 | 54.07 | 53.72 | 37.9 |

**Table S2.** FPKM values of pluripotency genes in wild-type and *Pten^−/−^* ESCs. Data related to Fig. 1f

**Table S3.** FPKM values of pluripotency genes in wild-type and Pten-A3 mutant ESCs. Data related to Fig. 5g

|  | **WT** | **WT** | **Pten-A3 mutant** | **Pten-A3 mutant** |
| --- | --- | --- | --- | --- |
| **Symbol** |  |  |  |  |
| *Nanog* | 477.85 | 478.32 | 233.84 | 342.4 |
| *Oct4* | 862.7 | 843.82 | 685.92 | 781.99 |
| *Klf4* | 86.44 | 91.3 | 49.41 | 68.2 |
| *Klf2* | 130.95 | 136.47 | 67.16 | 124.94 |
| *Nr0b1* | 56.08 | 56.1 | 38.88 | 43.88 |
| *Rex1* | 15.04 | 14.72 | 11.51 | 15.22 |
| *Fgf4* | 67.08 | 66.43 | 48.63 | 54.01 |
| *Lefty1* | 60.93 | 60.11 | 18.26 | 30.51 |
| *Ehmt2* | 31.13 | 31.28 | 26.74 | 32.18 |
| *Kdm5b* | 33.12 | 35.31 | 29.71 | 27.39 |
| *Sox15* | 15.32 | 15.96 | 7.94 | 9.05 |
| *E2f1* | 15.63 | 16.54 | 10.44 | 14.88 |
| *Wdr5* | 78.9 | 80.36 | 77.13 | 76.77 |
| *Gdf3* | 55.9 | 48.63 | 35.44 | 47.46 |
| *Cd9* | 209.03 | 195 | 162.54 | 176.21 |
| *Nodal* | 27 | 27.15 | 15.36 | 22.27 |
| *Ifitm2* | 532.17 | 526.21 | 297.38 | 313.92 |
| *Mycn* | 12.86 | 11.66 | 7.46 | 8.99 |

**Table S4.** FPKM values of pluripotency, ectoderm, endoderm, and mesoderm markers in wild-type and *Pten^−/−^* EBs. Data related to Fig. 6c

|  | **Lineage** | **WT EBs** | **WT EBs** | **WT EBs** | ***Pten^−/−^* EBs** | ***Pten^−/−^* EBs** | ***Pten^−/−^* EBs** |
| --- | --- | --- | --- | --- | --- | --- | --- |
| **Symbol** |  |  |  |  |  |  |  |
| *Nanog* | Pluripotency | 2.87 | 4.41 | 3.24 | 26.35 | 18.65 | 44.96 |
| *Oct4* | Pluripotency | 3.67 | 8.11 | 4.26 | 26.6 | 23.72 | 73.34 |
| *Esrrb* | Pluripotency | 0.4 | 0.18 | 0.28 | 2.85 | 2.43 | 7.53 |
| *Lin28b* | Pluripotency | 23.52 | 23.73 | 23.02 | 40.4 | 47.95 | 53.33 |
| *Lefty1* | Pluripotency | 0.57 | 0.57 | 0 | 3.29 | 2.01 | 6.48 |
| *Klf5* | Pluripotency | 1.1 | 1.61 | 1.17 | 4.33 | 3.43 | 6.69 |
| *Utf1* | Pluripotency | 0.55 | 1.21 | 0.28 | 6.25 | 5.62 | 12.71 |
| *Ctbp2* | Pluripotency | 21.78 | 26.46 | 25.85 | 28.77 | 27.9 | 35.43 |
| *Smarcc1* | Pluripotency | 70.43 | 76.35 | 75.6 | 87.12 | 81.76 | 79.87 |
| *Sp1* | Pluripotency | 26.63 | 24.96 | 25.15 | 29.61 | 30.37 | 31.41 |
| *Cetn2* | Pluripotency | 25.18 | 21.2 | 24.41 | 30.44 | 35.55 | 33.06 |
| *Dnmt3l* | Pluripotency | 0.5 | 0.32 | 0.26 | 6.53 | 7.08 | 18.21 |
| *Sox15* | Pluripotency | 0.18 | 0.09 | 0.09 | 1.84 | 1.7 | 3.61 |
| *Satb1* | Pluripotency | 6.23 | 6.05 | 5.86 | 8.68 | 9.32 | 7.64 |
| *Nodal* | Pluripotency | 0.6 | 1.58 | 1.62 | 5.82 | 2.87 | 4.4 |
| *Mycn* | Pluripotency | 11.51 | 15.45 | 14.92 | 34.12 | 34.13 | 39.7 |
| *Mycl* | Pluripotency | 0.63 | 2.01 | 1.24 | 5.48 | 7.1 | 10.67 |
| *Tet1* | Pluripotency | 6.15 | 5.49 | 5.62 | 9.67 | 9.36 | 13.83 |
| *Dppa3* | Pluripotency | 0.13 | 0 | 0.13 | 7.13 | 5.64 | 15.5 |
| *Gbx2* | Pluripotency | 0.15 | 0.05 | 0.25 | 2.77 | 2.64 | 3.82 |
| *Nes* | Ectoderm | 6.61 | 8.24 | 8.06 | 18.54 | 25.13 | 31.25 |
| *Pax6* | Ectoderm | 0.27 | 0.58 | 0.27 | 4.97 | 5.95 | 8.2 |
| *Fgf5* | Ectoderm | 0.09 | 1.71 | 0.56 | 2.23 | 1.82 | 6.48 |
| *Otx2* | Ectoderm | 0.58 | 2.18 | 0.39 | 7.14 | 6.8 | 15.54 |
| *Sall2* | Ectoderm | 9.65 | 10.88 | 9.34 | 21.48 | 22.5 | 22.32 |
| *Nefm* | Ectoderm | 0.13 | 0.23 | 0.33 | 1.87 | 3 | 3.9 |
| *Hoxa1* | Ectoderm | 1.84 | 2.01 | 1.63 | 6.9 | 5.11 | 4.79 |
| *Prom1* | Ectoderm | 1.99 | 2.2 | 2.29 | 13.87 | 12.97 | 15.67 |
| *Lef1* | Ectoderm | 4.36 | 7.91 | 7.01 | 14.75 | 13.5 | 11.46 |
| *Cxcl12* | Ectoderm | 23.77 | 14.33 | 18.49 | 29.87 | 30.71 | 29.35 |
| *Ascl1* | Ectoderm | 0 | 0.05 | 0 | 1.63 | 2.75 | 5.23 |
| *Sox1* | Ectoderm | 0 | 0.08 | 0 | 8.33 | 7.84 | 11.42 |
| *Hoxb1* | Ectoderm | 0.15 | 0.15 | 0.1 | 2.07 | 1.91 | 3.33 |
| *Map2* | Ectoderm | 0.59 | 0.5 | 0.54 | 2.2 | 2.51 | 5.05 |
| *Sox17* | Endoderm | 4.2 | 5.48 | 5.4 | 2.1 | 1.82 | 1.68 |
| *Gata4* | Endoderm | 14.98 | 18.22 | 13.86 | 14.99 | 13.65 | 10.08 |
| *Gata6* | Endoderm | 45.84 | 34.02 | 33.37 | 15.82 | 13.1 | 8.42 |
| *Hhex* | Endoderm | 16.98 | 15.71 | 14.13 | 5.02 | 4.71 | 4.05 |
| *Sox7* | Endoderm | 7.47 | 6 | 7.05 | 3.23 | 2.58 | 3.66 |
| *Emp2* | Endoderm | 18.97 | 17.75 | 18.23 | 8.23 | 5.61 | 1.59 |
| *Ihh* | Endoderm | 0.72 | 1.53 | 1.32 | 0.46 | 0.6 | 0.6 |
| *Spop* | Endoderm | 20.82 | 23.31 | 22.62 | 16.91 | 15.89 | 16.02 |
| *Hnf4a* | Endoderm | 1.4 | 3.22 | 2.25 | 0.92 | 1.2 | 1.06 |
| *Nkx2-5* | Mesoderm | 0.48 | 1.76 | 1.05 | 0.56 | 0.74 | 0.33 |
| *Gata3* | Mesoderm | 3.68 | 4.26 | 3.27 | 8.69 | 8.99 | 5.01 |
| *Tnnt2* | Mesoderm | 4.38 | 6.36 | 5.46 | 4.47 | 5.85 | 4.27 |
| *Brachyury* | Mesoderm | 0 | 1.04 | 0.57 | 0.31 | 1.05 | 2.79 |
| *Cebpa* | Mesoderm | 0.08 | 0.12 | 0.12 | 0.12 | 0.27 | 0.15 |
| *Cd34* | Mesoderm | 11.04 | 7.38 | 9.05 | 4.04 | 5.86 | 6.86 |
| *Shh* | Mesoderm | 0.31 | 0.39 | 0.43 | 0.65 | 1.6 | 2.42 |
| *Pitx2* | Mesoderm | 41.88 | 32.32 | 38.52 | 38.02 | 31.18 | 17.91 |
| *Elmo2* | Mesoderm | 12.74 | 11.21 | 10.4 | 13.41 | 11.67 | 11.78 |
| *Msx2* | Mesoderm | 16.1 | 22.79 | 18.82 | 30.42 | 22.31 | 12.39 |
| *Bmp4* | Mesoderm | 27.66 | 29.22 | 27.07 | 40.9 | 37.43 | 23.77 |
| *Tbx5* | Mesoderm | 2.16 | 1.92 | 1.62 | 2.03 | 1.58 | 0.69 |
| *Bmp2* | Mesoderm | 11.32 | 11.32 | 11.05 | 11.11 | 10.16 | 6.89 |

**Table S5.** FPKM values of naïve and primed pluripotency markers in wild-type and *Pten^−/−^* ESCs. Data related to Fig. S2a

|  | **Lineage** | **WT ESCs** | **WT ESCs** | **WT ESCs** | ***Pten^−/−^* ESCs** | ***Pten^−/−^* ESCs** | ***Pten^−/−^* ESCs** |
| --- | --- | --- | --- | --- | --- | --- | --- |
| **Symbol** |  |  |  |  |  |  |  |
| *Nanog* | Naïve | 284.71 | 231.25 | 213.47 | 430.91 | 409.68 | 273.49 |
| *Nr0b1* | Naïve | 28.02 | 37.38 | 36.79 | 50.15 | 54.99 | 42.78 |
| *Tfcp2l1* | Naïve | 66.45 | 32.24 | 32.4 | 96.65 | 96.24 | 44.16 |
| *Klf4* | Naïve | 58.05 | 53.79 | 54.03 | 88.08 | 80.03 | 72.49 |
| *Klf5* | Naïve | 117.08 | 121.55 | 122.76 | 142.42 | 130.25 | 115.36 |
| *Hormad1* | Naïve | 3.51 | 6.69 | 5.86 | 11.45 | 10.52 | 10.95 |
| *Tbx3* | Naïve | 42.01 | 22.6 | 22.09 | 58.02 | 52.36 | 33.57 |
| *Tet2* | Naïve | 44.5 | 35.33 | 33.08 | 54.07 | 53.72 | 37.9 |
| *Otx2* | Primed | 3.23 | 6.42 | 7.2 | 0.23 | 0.46 | 2.11 |
| *Sox4* | Primed | 8.78 | 13.49 | 14.39 | 4.13 | 3.59 | 6.39 |
| *Pou3f1* | Primed | 1.18 | 2.37 | 2.41 | 0.71 | 0.96 | 2.26 |
| *Dnmt3a* | Primed | 15.59 | 26.07 | 26.84 | 8.41 | 7.51 | 16.23 |
| *Lefty1* | Primed | 11.15 | 13.38 | 15.07 | 5.07 | 4.48 | 6.78 |
| *Lin28a* | Primed | 92.56 | 98.2 | 93.48 | 46.68 | 45.55 | 43.01 |

**Table S6.** Numbers of attached EBs vs suspended EBS. Data related to Fig. S3i

|  | **WT EBs** | | | ***Pten^−/−^* EBs** | | |
| --- | --- | --- | --- | --- | --- | --- |
| Attached EB | 14 | 10 | 8 | 7 | 10 | 6 |
| Suspended EB | 10 | 14 | 13 | 25 | 24 | 23 |
| Total number | 24 | 24 | 21 | 32 | 34 | 29 |
| Proportion of attached EBs | 58.33% | 41.67% | 38.09% | 21.88% | 29.41% | 20.69 |
